# Nicotinamide‐N‐methyltransferase is a promising metabolic drug target for primary and metastatic clear cell renal cell carcinoma

**DOI:** 10.1002/ctm2.883

**Published:** 2022-06-08

**Authors:** Anna Reustle, Lena‐Sophie Menig, Patrick Leuthold, Ute Hofmann, Viktoria Stühler, Christian Schmees, Michael Becker, Mathias Haag, Verena Klumpp, Stefan Winter, Florian A. Büttner, Steffen Rausch, Jörg Hennenlotter, Falko Fend, Marcus Scharpf, Arnulf Stenzl, Jens Bedke, Matthias Schwab, Elke Schaeffeler

**Affiliations:** ^1^ Dr. Margarete Fischer‐Bosch Institute of Clinical Pharmacology Stuttgart Germany; ^2^ University of Tuebingen Tuebingen Germany; ^3^ Department of Urology University Hospital Tuebingen Tuebingen Germany; ^4^ NMI Natural and Medical Sciences Institute at the University of Tuebingen Reutlingen Germany; ^5^ Experimental Pharmacology and Oncology GmbH Berlin‐Buch Germany; ^6^ Institute of Pathology and Neuropathology University Hospital Tuebingen Tuebingen Germany; ^7^ German Cancer Consortium (DKTK), Partner Site Tübingen German Cancer Research Center (DKFZ) Heidelberg Germany; ^8^ Departments of Clinical Pharmacology, Pharmacy and Biochemistry University of Tuebingen Tuebingen Germany; ^9^ Cluster of Excellence iFIT (EXC2180) ‘Image‐Guided and Functionally Instructed Tumor Therapies’ University of Tuebingen Tuebingen Germany

**Keywords:** glutamine, metabolism, metastasis, NNMT, NNMTi, oxphos, renal cell carcinoma

## Abstract

**Background:**

The metabolic enzyme nicotinamide‐N‐methyltransferase (NNMT) is highly expressed in various cancer entities, suggesting tumour‐promoting functions. We systematically investigated NNMT expression and its metabolic interactions in clear cell renal cell carcinoma (ccRCC), a prominent RCC subtype with metabolic alterations, to elucidate its role as a drug target.

**Methods:**

NNMT expression was assessed in primary ccRCC (*n* = 134), non‐tumour tissue and ccRCC‐derived metastases (*n* = 145) by microarray analysis and/or immunohistochemistry. Findings were validated in The Cancer Genome Atlas (kidney renal clear cell carcinoma [KIRC], *n* = 452) and by single‐cell analysis. Expression was correlated with clinicopathological data and survival. Metabolic alterations in NNMT‐depleted cells were assessed by nontargeted/targeted metabolomics and extracellular flux analysis. The NNMT inhibitor (NNMTi) alone and in combination with the inhibitor 2‐deoxy‐D‐glucose for glycolysis and BPTES (bis‐2‐(5‐phenylacetamido‐1,3,4‐thiadiazol‐2‐yl)ethyl‐sulfide) for glutamine metabolism was investigated in RCC cell lines (786‐O, A498) and in two 2D ccRCC‐derived primary cultures and three 3D ccRCC air–liquid interface models.

**Results:**

NNMT protein was overexpressed in primary ccRCC (*p* = 1.32 × 10^–16^) and ccRCC‐derived metastases (*p* = 3.92 × 10^–20^), irrespective of metastatic location, versus non‐tumour tissue. Single‐cell data showed predominant NNMT expression in ccRCC and not in the tumour microenvironment. High NNMT expression in primary ccRCC correlated with worse survival in independent cohorts (primary RCC—hazard ratio [HR] = 4.3, 95% confidence interval [CI]: 1.5–12.4; KIRC—HR = 3.3, 95% CI: 2.0–5.4). NNMT depletion leads to intracellular glutamine accumulation, with negative effects on mitochondrial function and cell survival, while not affecting glycolysis or glutathione metabolism. At the gene level, NNMT‐depleted cells upregulate glycolysis, oxidative phosphorylation and apoptosis pathways. NNMTi alone or in combination with 2‐deoxy‐D‐glucose and BPTES resulted in inhibition of cell viability in ccRCC cell lines and primary tumour and metastasis‐derived models. In two out of three patient‐derived ccRCC air–liquid interface models, NNMTi treatment induced cytotoxicity.

**Conclusions:**

Since efficient glutamine utilisation, which is essential for ccRCC tumours, depends on NNMT, small‐molecule NNMT inhibitors provide a novel therapeutic strategy for ccRCC and act as sensitizers for combination therapies.

## Graphical abstract

1. NNMT is highly expressed in ccRCC primary tumours and metastases, correlating with worse patient survival.
2. Glutamine metabolism is impaired in NNMT‐depleted cells, with negative consequences for cellular fitness.
3. NNMT inhibition reduces cell viability and induces cytotoxicity in 2D/3D ccRCC‐derived tumour models.
4. Beyond NNMT inhibition for the treatment of metabolic diseases, its application for anticancer therapy appears promising.

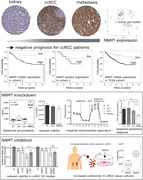

## 1 BACKGROUND

Nicotinamide‐N‐methyltransferase (NNMT) is a phase II metabolising enzyme that is mainly expressed in the liver but also in other organs, including the kidneys. It catalyses the transfer of a methyl group from S‐adenosylmethionine (SAM) to a broad range of substrates, forming S‐adenosyl‐homocysteine and the methylated substrate. With this methylation reaction, NNMT facilitates the excretion of its substrates, which was long considered the main function of NNMT. However, studies in recent years have shown increased expression of NNMT in various types of cancer, including clear cell renal cell carcinoma (ccRCC),^1^, ^2^ implying functions of this enzyme in tumour development and/or growth.^3^ The mechanisms of increased expression of NNMT in cancer are not completely understood; however, induction by hepatocyte nuclear factor‐1β (HNF1β),^4^ signal transducers and activators of transcription 3 (STAT3),^5^ or transforming growth factor‐β (TGFβ)^6^ appears to be involved. Similarly, the functions proposed for NNMT in cancer are diverse. High NNMT expression was shown to increase replication,^7^ migration and invasion,^8^, ^9^ as well as adenosine triphosphate (ATP) synthesis^10^, ^11^ in cancer cells. Moreover, by consuming SAM and generating a methylation sink within cells, NNMT inhibits the activities of other methyltransferases, leading to decreased histone and DNA methylation and consequently altered gene expression in cancer cells.^12^, ^13^ Furthermore, NNMT withdraws nicotinamide, its preferred substrate, from the nicotinamide adenine dinucleotide (NAD^+^) salvage pathway that provides NADH for mitochondrial energy production. In ccRCC, NNMT‐derived peptides are human leucocyte antigen (HLA) dependently presented on the surface of tumour cells.^14^ Taken together, NNMT might have miscellaneous effects on cancer cell metabolism that act together to support tumourigenesis and invasive cancer growth.

The entanglement of NNMT with cellular metabolism makes it particularly interesting for a comprehensive study in ccRCC, the most common subtype of renal cancer. ccRCC can be considered a metabolic disease, since loss of chromosome arm 3p and inactivation of the Von‐Hippel Lindau (VHL) tumour suppressor and the resultant constitutively active hypoxia‐inducible factor (HIF) transcription factor in the majority of tumours lead to marked metabolic changes.^15^, ^16^, ^17^ These changes include upregulation of aerobic glycolysis to form lactate (Warburg effect), the pentose phosphate pathway, fatty acid synthesis, glutamine and glutathione (GSH) metabolism, and downregulation of the tricarboxylic acid (TCA) cycle, fatty acid β‐oxidation and oxidative phosphorylation (oxphos).^18^ In addition, constitutive activation of HIF imitates a hypoxic environment and induces the formation of new blood vessels (neo‐angiogenesis) in the growing tumour. Antiangiogenic agents, together with inhibitors of the mechanistic target of rapamycin (mTOR) pathway, that are affected by activating mutations in ccRCC, and immune checkpoint inhibitors are therefore used in the clinical management of advanced and metastatic ccRCC.^19^, ^20^ Initial response rates of single agents range approximately 30%, and usually patients are treated in several treatment lines with agents from the different classes. Nevertheless, due to resistance development and tumour recurrence, metastatic ccRCC is rarely cured, and only 13% of those patients survive 5 years or more after diagnosis.^21^ To increase response rates and to extend the survival of patients with advanced or metastatic ccRCC, new drug targets from alternative cellular pathways that can be combined with already existing therapies are urgently needed.

Therefore, we investigated NNMT in primary ccRCC and ccRCC‐derived metastasis in an effort to better understand its role in tumour metabolism and to elucidate its eligibility as a metabolic drug target. For in vitro experiments, we used the established ccRCC‐derived cell lines 786‐O and A498 and two newly established ccRCC‐derived primary cultures (RCC1 and RCC2). In addition, ex vivo 3D air–liquid interface (ALI) models^22^ of ccRCC were used to test the NNMT inhibitor 5‐amino‐1‐methylquinolium (NNMTi).^23^

## 2 METHODS

### 2.1 Patient cohorts

We studied NNMT expression in two primary ccRCC cohorts and one cohort comprising ccRCC‐derived metastases (Table 1). Cohort 1 contained 134 ccRCC tissues that were treated at the Department of Urology, University Hospital Tuebingen, Germany. A second cohort consisted of 145 ccRCC‐derived metastases from 78 patients (cohort 2), collected again at the Department of Urology, University Hospital Tuebingen, Germany. For these patients, formalin‐fixed and paraffin‐embedded tissues were available for 64 primary ccRCC and 50 paired non‐tumour tissue samples (cohort 1), as well as all 145 metastatic ccRCC sections of cohort 2. Fresh‐frozen tissues from cohort 1 (*n* = 124) were available for mRNA expression analysis. Clinicopathological data for both cohorts were collected at the Department of Urology at the University Hospital Tuebingen and evaluated independently by at least two urologists. Data were analysed retrospectively in this study. In addition, the kidney renal clear cell carcinoma (KIRC) cohort of The Cancer Genome Atlas (TCGA KIRC cohort), containing 452 ccRCC tissues and 67 paired non‐tumour tissues, was analysed. Clinical and transcriptome data were downloaded from the Genomic Data Commons Portal.^24^

**TABLE 1:** Detailed description of clear cell renal cell carcinoma (ccRCC) patient cohorts

	Cohort 1: primary ccRCC		Cohort 2: metastases		TCGA KIRC cohort	
	No. of patients	%	No. of patients	%	No. of patients	%
No. of patients	134^a^		78^b^		452^c^	
Sex						
Male	83	61.9	60	76.9	290	64.2
Female	51	38.1	18	23.1	162	35.8
Age median (range)	65 (35–90)		61 (29–77)		61 (29–90)	
Stage						
NA	–	–	6	7.7	–	–
1	55	41.0	13	16.7	221	48.9
2	9	6.7	8	10.3	44	9.7
3	42	31.3	30	38.5	117	25.9
4	28	20.9	21	26.9	70	15.5
Primary tumour						
NA	–	–	7	9.0	–	–
1	60	44.8	19	24.4	227	50.2
2	10	7.5	10	12.8	56	12.4
3	62	46.3	40	51.3	164	36.3
4	2	1.5	2	2.6	5	1.1
N						
NA	1	0.7	7	9.0	‐	‐
X	6	4.5	1	1.3	238	52.7
0	113	84.3	63	80.8	203	44.9
1/2	14	10.4	7	9.0	11	2.4
M						
NA	–	–	1	1.3	–	–
X	1	0.7	‐	‐	7	1.5
0	106	79.1	56	71.8	377	83.4
1	27	20.1	21	26.9	68	15
G						
NA	2	1.5	9	11.5	2	0.4
X	–	–	–	–	1	0.2
1	24	17.9	8	10.3	10	2.2
2	81	60.4	36	46.2	188	41.6
3	27	20.1	25	32.1	185	40.9
4					66	14.6
Follow‐up median (range)	5.1 (0.0–10.5)		7.8 (0.4–30.4)		3.5 (0–12.4)	
Overall survival						
Alive	85	63.4	23	29.5	307	67.9
Dead	48	35.8	55	70.5	145	32.1
Cancer‐specific survival^d^						
Alive/non‐cancer‐related death	97	72.4	25	32.1	354	78.3
Cancer‐related death	34	25.4	53	67.9	87	19.2

Abbreviations: G, nuclear grade; M, distant metastases; N, lymph node metastases; NA, not available; TCGA KIRC, The Cancer Genome Atlas kidney renal clear cell carcinoma.

^a^ Cohort comprises 50 corresponding non‐tumour tissues.

^b^ Cohort includes 145 metastases, since for some patients multiple metastases in different organs were analysed. Tumour stage, primary tumour characteristics, lymph node metastases, distant metastases and grading refer to the initial tumour diagnosis.

^c^ The cohort comprises 67 corresponding non‐tumour tissues.

^d^ Data were not available for all patients.

### 2.2 NNMT immunohistochemical staining

Tissue microarrays were processed as described.^25^ NNMT protein was stained with a monoclonal antibody (Santa Cruz Biotechnology Cat# SC‐376048) at a 1:50 dilution. The stained slides were scanned with an SCN400 slide scanner (Leica Microsystems). Cellular NNMT staining was analysed with the software‐based image analysis system TissueStudio (Definiens AG). The mean chromogen intensities in the tumour and non‐tumour areas were used as NNMT expression values. If more than one core was available per tissue, the mean expression was calculated. The antibody used in immunohistochemical (IHC) was validated by siRNA knockdown and Western blot (Figure S1A). Knockdown was performed as described below. For Western blotting, cells were harvested in sodium phosphate buffer containing pefabloc (Carl Roth Cat# A154.1) and protease inhibitor cocktail (Sigma, Cat# P8340). Cells were lysed by sonication, and membrane and cytoplasmic components were separated by centrifugation. For Western blotting, 10 μg of total protein was loaded per lane. Proteins were transferred to a nitrocellulose membrane by semidry transfer. Primary NNMT antibody was diluted 1:100 in 3% skim milk in Tris‐buffered saline with Tween 20 (TBST) and incubated at 4°C overnight. The β‐actin antibody (Sigma Cat# A4551) and secondary antibody (Santa Cruz Biotechnology Cat# sc‐2031) were diluted 1:5000 in 3% skim milk in TBST and incubated for 1 h at room temperature.

### 2.3 mRNA extraction and analysis

Tissue mRNA was extracted and analysed by HTA 2.0 microarrays (Thermo Fisher Scientific Cat# 902162) as previously described.^26^, ^27^ Array quality control was conducted by Affymetrix Expression Console (Build 1.4.1.46; Thermo Fisher Scientific). Microarrays were normalised individually using the SCAN method from the R package SCAN.UPC (version 2.26.0).^28^ Probe sets were summarised on the Entrez GeneID level using the annotation provided by BrainArray (version 23).^29^, ^30^ mRNA of cultured cells was extracted with the mirVana miRNA isolation kit (Thermo Fisher Scientific Cat# AM1560) and analysed by HTA 2.0 microarrays. Quality checks and robust multichip average (RMA) normalisation were carried out as previously described.^26^, ^27^ HTA 2.0 Transcript Cluster IDs were assigned to gene symbols and Entrez Gene IDs with the Affymetrix Annotation file provided by the NetAffx Analysis Center.^31^

### 2.4 Analysis of single‐cell gene expression data

The single‐cell gene expression data set from Young et al.^32^ was downloaded from the paper's supplement and analysed and visualised in R/RStudio^33^, ^34^ with the additional packages Seurat (version 4.0.3),^35^ sva (version 3.38.0), scran (version 1.18.7), org.Hs.eg.db (version 3.12.0), plyr (version 1.8.6) and ggplot2 (version 3.3.5). Cluster annotation was achieved manually and with the SingleR package (version 1.8.1).^36^

### 2.5 Cell culture

786‐O cells (CLS Cat# 300107/p747_786‐O) were cultured in Roswell Park Memorial Institute (RPMI) medium 1640 (Lonza Cat# BE12‐167F) supplemented with 10% foetal calf serum (FCS; Sigma Cat# F7524) and 2 mM L‐glutamine (Lonza Cat# BE17‐605E) at 37°C and 5% CO_2_. A498 cells (CLS Cat# 300113) were cultured in Eagle's Minimal Essential Medium (EMEM) (Lonza Cat# BE12‐125F) supplemented with 10% FCS and 2 mM L‐glutamine. Cell line authentication was performed using the PowerPlex 21 System (Promega). Routine testing for mycoplasma infection was performed with a PCR‐based test (Venor GeM Classic, Minerva Biolabs GmbH). One 2D ccRCC‐derived primary cell culture model (RCC1) was derived from a lymph node metastasis of a male patient with ccRCC features and multiple metastases. The second RCC2 model was derived from a primary ccRCC tumour of a female patient. Primary models were generated in collaboration with the NMI Natural and Medical Science Institute at the University of Tuebingen, Germany and EPO (Experimental Pharmacology & Oncology Berlin‐Buch GmbH). Primary cells were cultured in the same media and atmospheric conditions as 786‐O cells, with the addition of 100 U/ml penicillin/streptomycin (Thermo Fisher Scientific Cat# 11548876).

### 2.6 NNMT knockdown

Knockdown was achieved with a mixture of four siRNA sequences targeting NNMT mRNA (Horizon Discovery Cat# M‐010351‐01). Two nontargeting siRNA pools (ctr.1: Horizon Discovery Cat# D‐001206‐13, ctr.2: Horizon Discovery Cat# D‐001810‐10) and mock‐transfected cells (untreated [UT]) served as controls. Cells were transfected with the DharmaFECT 1 transfection reagent (Horizon Discovery Cat# T‐2001‐01) according to the manufacturer's instructions. Knockdown efficiency was assessed by quantitative real‐time PCR with specific assays for NNMT (Thermo Fisher Scientific, Cat# Hs00196287_m1) and β‐actin (Thermo Fisher Scientific, Cat# 10331085). The knockdown efficiency was generally >90% in the experiments (Figure S1B,C).

### 2.7 Metabolomics analysis

For metabolomics analysis, approximately 1 × 10^6^ cells were harvested 48 h after transfection. Four hours prior to harvest, the culture medium was exchanged with Opti‐MEM reduced‐serum medium (Thermo Fisher Scientific Cat# 51985026). Cells were detached with StemPro Accutase (Thermo Fisher Scientific Cat# A1110501) and collected by centrifugation at 4°C. Pellets were flash‐frozen in liquid nitrogen and kept at ‐80°C until metabolite extraction. Metabolites were extracted using 80% organic solvent as described.^37^, ^38^ In brief, pellets were taken up in 150 μl ice cold methanol:acetonitrile:water (2:2:1, v/v/v) and lysed by three cycles of flash‐freezing, thawing and ultrasonic treatment. Afterwards, lysates were frozen for 1 h at ‐20°C, and debris was removed by centrifugation. The remaining supernatant was transferred to a new vial and dried by evaporation with N_2_ gas. For nontargeted metabolomics analysis, extracts were reconstituted in 100 μl water:acetonitrile (5:95, v/v) and analysed by hydrophilic interaction liquid chromatography on a 1290 Infinity ultrahigh‐performance liquid chromatography system coupled to a 6550 iFunnel quadrupole time‐of‐flight mass spectrometer (LC‐QTOF‐MS) from Agilent Technologies as previously described.^39^ For targeted analysis, pellets were resuspended in 50 μl of methanol:water (1:1, v/v). Intracellular and extracellular concentrations of pyruvate, fumarate, malate, α‐ketoglutarate, hydroxyglutarate, citrate and proteinogenic amino acids were determined by gas chromatography–mass spectrometry as described previously.^40^, ^41^ Ornithine and citrulline were quantified by liquid chromatography with tandem mass spectrometry (LC–MS–MS) analysis similar to a published method.^42^ Briefly, 10 μl of cell culture supernatant or 5 μl of cell extract was added to a mixture of 10 μl of internal standard (25 pmol/μl each of [2H7]citrulline and [2H7]ornithine), 10 μl of water and 20 μl of mobile phase A (see below). After centrifugation, 1 μl of the supernatant was used for LC–MS–MS analysis. High‐performance liquid chromatography gradient elution was performed on a ZorbaxSB C‐18 column (2.1 × 50 mm, 1.8 μm particle size, Agilent). The mobile phases consisted of water (A) and acetonitrile (B), each containing 0.1% heptafluorobutyric acid and 0.5% formic acid. Ionisation mode was electrospray, polarity positive. The mass spectrometer was operated in multiple reaction monitoring (MRM) mode using the following MRM transitions: 133.1 → 70.2 for ornithine, 140.1 → 77.1 for [2H7]ornithine, 176.1 → 70.1 for citrulline and 183.1 → 77.1 for [2H7]citrulline. Data were analysed in R/RStudio^33^, ^34^ with the additional packages lumi (version 2.36.0), Hmisc (version 4.4.1) and beeswarm (version 0.2.3). For nontargeted analysis, feature intensities were normalised to the sum of all feature intensities in a given sample.

### 2.8 Cell viability assay

Cell viability was assessed with the RealTime‐Glo MT Cell Viability Assay (Promega Cat# G9711) according to the manufacturer's instructions.

### 2.9 Glutathione assay

Levels of reduced and oxidised glutathione (GSSG) were quantified with the GSH/GSSG‐Glo Assay (Promega Cat# V6611) according to the manufacturer's instructions.

### 2.10 Extracellular flux analysis

Extracellular flux analysis was performed with the Agilent Seahorse Xfe96 Analyser. Glycolysis (Agilent Cat# 103020‐100) and Mitochondrial Stress Test (Agilent Cat# 103015‐100) kits were used to assess glycolysis and mitochondrial function, respectively. The Mito Fuel Flex Test (Agilent Cat# 103260‐100) was used to assess glucose, fatty acid and glutamine dependencies. Data were analysed with Seahorse Wave 2.6 (Agilent, RRID:SCR_014526) and R/RStudio.^33^, ^34^

### 2.11 Gene set enrichment analysis

Gene set enrichment analysis (GSEA) was carried out in R/RStudio^33^, ^34^ with the additional packages GSVA (method ‘ssgsea’, version 1.32.0)^43^ and piano (version 2.0.2).^44^ For piano GSEA, fold‐chances between NNMT_kd_ and ctr.1 cells were used as input, and the ‘gsea’ method was chosen as gene set statistics (geneSetStat). Hallmark and glutamine‐related gene signatures were retrieved from the molecular signatures database MsigDB.^45^

### 2.12 NNMT inhibition

Both ccRCC cell lines (786‐O, A498) and ccRCC‐derived primary and metastatic (RCC1, RCC2) models were treated with the NNMT inhibitor NNMTi (R&D Systems Cat# 6900) alone or in combination with 2‐deoxy‐D‐glucose (2‐DG; Sigma–Aldrich Cat# D6134) or BPTES (Selleckchem Cat# S7753). Cell viability was monitored continuously with the RealTime‐GloTM MT Cell Viability Assay (Promega Cat# G9711) according to the instructions. Alternatively, cells were harvested for metabolomic analysis 24 h after inhibitor treatment. To assess NNMT inhibitor activity ex vivo, we used patient‐derived 3D ALI models of three ccRCC patients, as described by Neal et al.^22^ Treatment‐naïve tumour tissues were provided by the Department of Urology at the University Hospital Tuebingen, located in Tuebingen, Germany. In brief, tumour tissues were finely minced into approximately 2 × 2 mm pieces and resuspended in type I collagen gel (R&D Systems Cat# 3447‐020‐01). The tumour–collagen mixture was placed on top of a solidified collagen gel within a transwell insert containing a permeable membrane at the bottom. The insert was placed into a culture dish containing advanced Dulbecco's Modified Eagle Medium/Nutrient Mixture F‐12 (DMEM/F12) medium (Gibco) supplemented with R‐spondin (100 ng/ml, Peprotech), N‐2‐hydroxyethylpiperazine‐N'‐2‐ethanesulfonic acid (HEPES; 10 mM, Sigma), GlutaMAX (1×, Gibco), nicotinamide (10 mM, Sigma), N‐acetylcysteine (1 mM, Sigma), B27 supplement without vitamin A (1×, Gibco), A83‐01 (0.5 μM, Tocris), gastrin (10 nM, Sigma), SB202190 (10 μM, Stemcell), epidermal growth factor (EGF; 50 ng/ml, Peprotech), Noggin (25 ng/ml, Peprotech), interleukin 2 (IL‐2; 600 units/ml, Peprotech) and penicillin/streptomycin (100 units/ml, Gibco). ALIs were treated with 100 μM NNMTi or dimethyl sulfoxide (DMSO) in duplicate or triplicate wells. After 24 h, cytotoxicity was assessed by CellTox Green reagent (Promega Cat# G8741) according to the manufacturer's instructions. Z‐stack images were taken for each well with the Cytation 1 imager (Agilent) and merged in ImageJ software.^46^ Regions of interest (ROIs) were placed around tissues, and the integrated density was calculated for all tissue pieces per well. For each well, background correction was performed by subtracting the integrated density of ROIs without tissue. Background‐corrected intensities were plotted in R with the ggplot2 package.

### 2.13 Flow cytometry

Naïve T cells were generated from peripheral blood mononuclear cells of healthy volunteers by stimulation with anti‐CD3/anti‐CD28 beads (Thermo Fisher Scientific Cat# 11161D) and IL‐2 (200 units/ml, Peprotech) for 48 h in advanced RPMI 1640 (Gibco) supplemented with L‐glutamine (2 mM, Lonza), MEM vitamins (1×, Gibco), human serum (5%, Sigma) and primocin (0.1 mg/ml, Cayla‐InvivoGen). After magnetic bead separation, T cells were treated for 72 h with 10 mM 1‐methyl‐nicotinamide (1‐MNA; Sigma, Cat# M4627) alone or in combination with 2 mg/ml TGFβ (Peprotech Cat# 100‐21C). Half of the cells were additionally stimulated with 20 ng/ml phorbol 12‐mistrate 13‐acetate (PMA; Peprotech Cat# 1652981) and 1 μg/ml ionomycin (Peprotech Cat# 5608212) 4 h prior to analysis. For surface antigen staining, 1 × 10^6^ cells were harvested and blocked with an Fc receptor blocking solution (Biolegend Cat# 422302). Dead cells were stained with the LIVE/DEAD Fixable Aqua Dead Cell Stain Kit (Thermo Fisher Scientific Cat# L34966). The following primary antibodies were used in the experiment: allophycocyanineAPC antihuman CD3 (Biolegend Cat# 317318), APC/cyanine7 antihuman CD4 (Biolegend Cat# 344616), Brilliant Violet 605 antihuman CD8a (Biolegend Cat# 300936), PerCP/cyanine5.5 antihuman CD152 (Biolegend Cat# 369607) and Pacific Blue antihuman CD279 (Biolegend Cat# 329915). Corresponding isotype controls were also purchased from Biolegend. For flow cytometric analysis, a FACSLyric (BD Biosciences) instrument was used. Data were collected and visualised with FACSuite software (BD Biosciences) and analysed in R and RStudio.

### 2.14 Data analysis and statistics

Analyses of data from cell culture experiments were performed in R/RStudio^33^, ^34^ with the additional packages beeswarm (version 0.2.3) and ggplot2 (version 3.3.5) and in GraphPad Prism (GraphPad Software Inc., version 5.04). In the nontargeted metabolomics experiment, we applied Welch's tests for comparisons between NNMT_kd_ cells and each of the three controls (Table S1). The unadjusted *p*‐values were used as criteria to select significantly regulated metabolites in NNMT knockdown cells compared to controls. Information regarding the adjustment of *p*‐values by the Benjamini–Hochberg method to correct for multiple testing is given in Table S1. Data from the targeted metabolomics analysis experiment and the GSH assay were investigated by repeated measures analysis of variance and post hoc Tukey's range test. For extracellular flux analyses and inhibitor experiments, we calculated the pooled standard error of replicate experiments and used the pt() function in R to calculate corresponding *p*‐values. Here, *p*‐values were corrected for multiple testing with the Benjamini–Hochberg method. For clinical data, Wilcoxon signed rank and rank sum tests were used to check for differences in paired and unpaired samples, respectively. Survival analysis was performed with the packages party (version 1.3‐5) and survival (version 3.2‐7) in R/RStudio. Optimal cut‐offs and corresponding *p*‐values were determined by conditional interference tree models (ctree function). Cox proportional hazard regression models were used to estimate hazard ratios (HR) and corresponding confidence intervals. Survival functions were estimated by Kaplan–Meier curves. All statistical tests were two sided, and the significance level was set to 5%.

## 3 RESULTS

### 3.1 NNMT is highly expressed in primary ccRCC and metastases and associated with inferior patient survival

**FIGURE 1:**
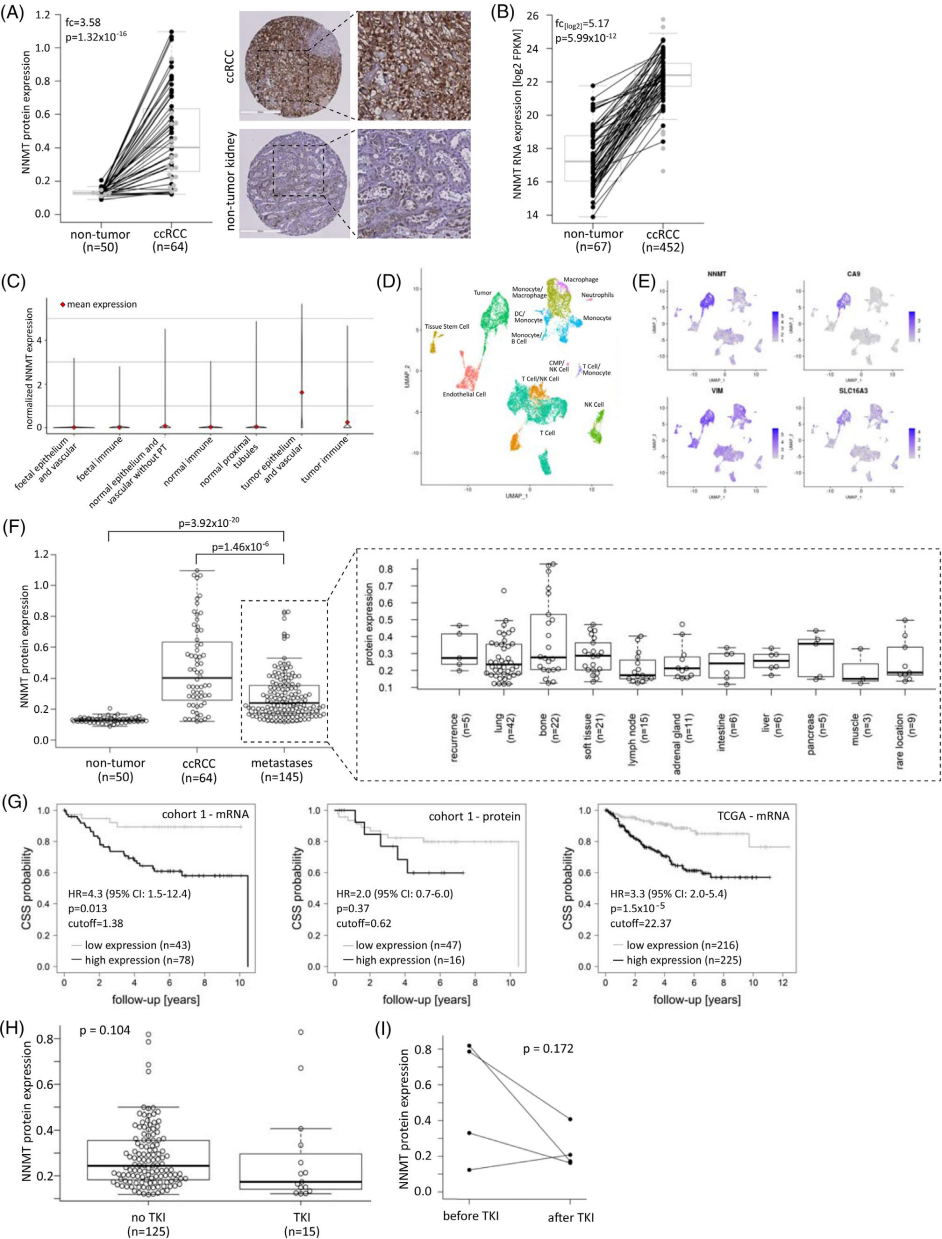
Nicotinamide‐N‐methyltransferase (NNMT) expression and association with survival in clear cell renal cell carcinoma (ccRCC) patient cohorts. (A) Analysis of NNMT protein expression in tumour and non‐tumour tissues of patient cohort 1 (*n* = 64, left panel). Paired samples are connected by lines. Grey dots represent the expression values of unpaired samples. The fold‐change (fc) and Wilcoxon rank sum *p*‐value of the unpaired analysis are given in the plot. An example of non‐tumour and ccRCC NNMT immunohistochemical staining is shown on the right. (B) NNMT mRNA expression in tumour and non‐tumour samples of The Cancer Genome Atlas (TCGA) ccRCC patient cohort (*n* = 452). Paired samples are connected by lines. Grey dots represent outliers of the unpaired analysis. The fc and Wilcoxon rank sum *p*‐values of the unpaired analysis are given in the plot. (C) The highest expression of NNMT was shown in the tumour epithelium and vascular compartment using single‐cell gene expression data.^32^ (D) Clustering of single‐cell data of two RCC samples, performed with the Seurat package in R and manually or with the help of the SingleR package annotated, revealed tumour cells, endothelial cells, tissue stem cells and various immune cell populations. (E) Expression of NNMT and the ccRCC tumour markers carbonic anhydrase 9 (CA9), vimentin (VIM) and monocarboxylate transporter 4 (SLC16A3) are shown in the clusters from panel 1D, indicating the highest NNMT expression in the tumour cell cluster. (F) NNMT protein expression in non‐tumour tissue (cohort 1, *n* = 50), primary ccRCC (cohort 1, *n* = 64) and ccRCC‐derived metastases (cohort 2, *n* = 145). Wilcoxon rank sum *p*‐values for fc between non‐tumour and metastatic tissues (fc = 2.14) and ccRCC and metastatic tissues (fc = 0.60) are given in the plot. Rare metastatic locations include the skin, thyroid, larynx, parotid gland, nerve, uterus, spleen and testis. (G) Cancer‐specific survival of ccRCC patients with high versus low NNMT expression (mRNA, *n* = 121; protein, *n* = 63) in primary ccRCC (cohort 1) and the TCGA KIRC cohort (*n* = 441). Cut‐offs, hazard ratios (HR) and *p*‐values as determined by conditional interference tree models (see Section 2) are given in the plots. (H) NNMT expression in metastases of patients untreated (*n* = 125) or pretreated (*n* = 15) with tyrosine kinase inhibitors (TKIs). The Wilcoxon rank sum *p*‐value is given in the plot. (I) NNMT expression in metastases from four patients before and after TKI treatment. The Wilcoxon signed rank *p*‐value is given

NNMT protein assessed by IHC staining was significantly more highly expressed in primary ccRCC than in non‐tumour tissue of cohort 1, in unpaired (Figure 1A, *n* = 64, fold‐changes [fc] = 3.58, *p* = 1.32 × 10^–16^) as well as in paired samples (Figure 1A, *n* = 44, fc = 3.96, *p* = 3.41 × 10^–13^). A significant correlation between mRNA and protein expression was found for the ccRCC samples (Spearman correlation, *R* = 0.30, *p* = .03). Higher mRNA expression was also found for tumour samples of the TCGA KIRC cohort compared to non‐tumour tissue in unpaired (Figure 1B, *n* = 452, fc_[log2]_ = 5.17, *p* = 5.99 × 10^–12^) as well as in paired samples (Figure 1B, *n* = 67, fc_[log2]_ = 4.93, *p* = 1.15 × 10^–12^). High NNMT expression in primary ccRCC correlated with advanced disease grade (*p* = .030), stage (*p* = .022) and the presence of distant metastases (*p* = .006) in the TCGA KIRC cohort (*n* = 452) but not in primary ccRCC cohort 1 (Figure S2).

Analysis of a publicly available single‐cell gene expression data set^32^ showed the highest expression of NNMT in the tumour epithelium and vascular compartment compared to foetal and adult normal kidney compartments and the normal and tumour immune compartments (Figure 1C). Gene expression‐based clustering of single cells and cluster annotation in two RCC samples part of the study by Young et al. revealed tumour cells, endothelial cells, tissue stem cells and various immune cell populations in the RCC samples (Figure 1D). NNMT was again most highly expressed in the tumour cell cluster, confirming its prominent expression in ccRCC tumour tissue (Figure 1E).

NNMT protein was also highly expressed in ccRCC‐derived metastases (cohort 2, *n* = 145), with bone and lung metastases showing the highest expression levels (Figure 1F). Metastatic NNMT expression was significantly higher than that in non‐tumour tissue (Figure 1F, fc = 2.15, *p* = 3.92 × 10^–20^) and slightly lower than that in primary RCC (Figure 1F, fc = 0.60, *p* = 1.46 × 10^–6^). From 44 patients in our metastasis cohort, we had more than one metastatic tissue available. Analysis of these tissues showed that NNMT protein expression varied within metastases from the same patient, independent of metastasis location or time after initial cancer diagnosis (Figure S3A). Furthermore, cohort 2 included four pairs of metastases representing metastatic progression. In two of those cases, NNMT protein expression was higher in the progressed metastasis than in the initially resected metastasis (Figure S3B). Generally, there was no increase in NNMT protein expression in metastases diagnosed at later versus earlier time points after the initial cancer diagnosis (Figure S3C).

Regarding patient outcome, importantly, high NNMT mRNA expression significantly correlated with worse patient survival in cohort 1 (HR = 4.3, 95% confidence interval [CI]: 1.5–12.4), which was confirmed in the TCGA KIRC cohort (HR = 3.3, 95% CI: 2.0–5.4) (Figure 1G). The same trend was also observed for NNMT protein expression in cohort 1, although the difference did not reach statistical significance (HR = 2.0, 95% CI: 0.7–6.0), likely due to the limited number of samples in this analysis (Figure 1G).

We did not observe altered NNMT protein expression in metastases pretreated with tyrosine kinase inhibitors (TKIs) (Figure 1H), the most frequently applied systemic therapy in our metastatic patient cohort 2. In three of four patients with metastases resected before and after systemic therapy, NNMT expression was lower after TKI treatment (Figure 1I). An overview of systemic treatments in cohort 2 is given in Table 2.

**TABLE 2:** Systemic pretreatment in metastatic clear cell renal cell carcinoma (ccRCC) patients (cohort 2)

Type	Substance	No. of metastases with pretreatment
TKI	Sunitinib	6
mTOR	Temsirolimus	2
Other	PEG‐glutaminase + DON	2
	Roferon A/vinblastin	3
Sequence	TKI	4
	TKI—mTOR	2
	TKI—other	3
None		118
No information		5

Abbreviations: DON, 6‐diazo‐5‐oxo‐L‐norleucine; mTOR, mechanistic target of rapamycin inhibitor; PEG, pegylated; TKI, tyrosine kinase inhibitor.

### 3.2 NNMT knockdown affects glutamine metabolism and cell viability in RCC cells

**FIGURE 2:**
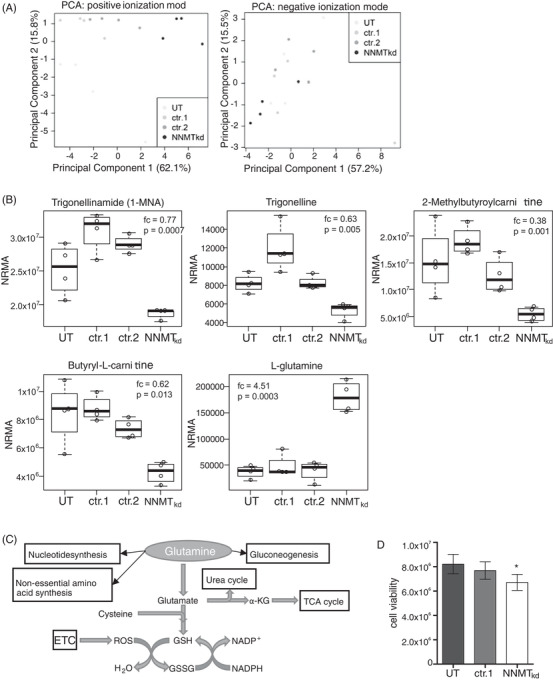
Nontargeted metabolomics analysis. (A) Principal component analysis (PCA) of detected features in positive (left panel) and negative (right panel) ionisation modes of the nontargeted metabolomics analysis. (B) Differentially abundant metabolites in nicotinamide‐N‐methyltransferase knockdown (NNMT_kd_) cells and untreated control cells (UT) or cells transfected with nontargeting siRNA control pools (ctr.1, ctr.2). Fold‐changes (fc) and *p*‐values (Student's *t*‐test with Welch correction for unequal variances) compared to control siRNA 1 (ctr.1)‐transfected cells are given in the graphs. The values represent the normalised relative metabolite abundances (NRMA). (C) Schematic view of glutamine utilisation in cell metabolism. (D) Cell viability of NNMT_kd_ and control cells. Viability was measured 48 h after NNMT knockdown. Values represent the mean values ± standard deviation of three independent experiments. Student's *t*‐test was used to compare cell viability in NNMT_kd‐_ and ctr.1‐transfected cells (*p < .05)

To investigate metabolic changes that may occur in cells with depleted NNMT expression, we chose a nontargeted metabolomics approach. Therefore, we performed siRNA‐mediated knockdown of NNMT in the 786‐O renal cell carcinoma cell line (NNMT_kd_) and analysed the cell lysates by LC‐QTOF‐MS. Of all detected features, 35 features were differentially regulated (fc > 1.2, *p* < .05) in NNMT_kd_ cells compared to controls (Table S1). Furthermore, cells with depleted NNMT expression formed a separate cluster when analysed by principal component analysis based on all detected features in positive mode (Figure 2A, left panel) but not in negative mode (Figure 2A, right panel). Five of the differentially regulated features could be assigned to defined metabolites (level of assignment [LoA] ≥ 2), including 1‐MNA (fc = 0.77, *p* = .0007), which is a direct product of the metabolism of nicotinamide by NNMT. The other deregulated metabolites were trigonelline (fc = 0.63, *p* = .005), 2‐methylbutyroylcarnitine (fc = 0.38, *p* = .001), butyryl‐L‐carnitine (fc = 0.62, *p* = .013) and, most prominently, the amino acid L‐glutamine (fc = 4.51, *p* = .0003), further referred to as glutamine (Figure 2B). Glutamine is a substrate for various cellular pathways, ranging from DNA/RNA and protein synthesis to energy and GSH metabolism (Figure 2C). Notably, ccRCC relies on glutamine for tumour growth,^47^ which has led to the investigation of the glutaminase (GLS) inhibitor CB‐839 in a phase I clinical study with patients with advanced/metastatic ccRCC.^48^ The finding of increased levels of glutamine in NNMT_kd_ cells raised the question of whether NNMT might impact glutamine metabolism in ccRCC, with potential consequences on the fitness and survival of tumour cells. To assess the effect of NNMT knockdown on the fitness of 786‐O cells, we analysed cell viability. Indeed, viability in NNMT_kd_ cells was decreased by 18.3% compared to UT controls and by 12.7% compared to nontargeting siRNA‐transfected control cells (NNMT_kd_ vs. ctr.1, *p*_(t‐test)_ = .0315) (Figure 2D).

### 3.3 Targeted metabolomics analysis of amino acids and TCA cycle intermediates confirms the accumulation of glutamine in NNMT_kd_ cells

**FIGURE 3:**
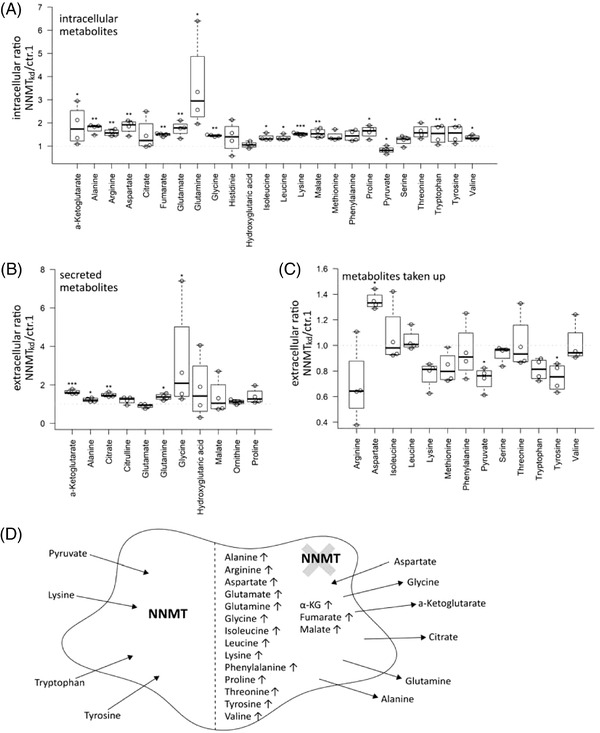
Targeted metabolomics analysis. (A) Ratios of intracellular metabolite concentrations in nicotinamide‐N‐methyltransferase knockdown (NNMT_kd_) cells versus cells transfected with control siRNA 1 (ctr.1). (B) Ratios of secreted metabolites in NNMT_kd_ and ctr.1 cells. (C) Ratios of metabolite uptake in NNMT_kd_ and ctr.1 cells. The boxplots show median values and 25% and 75% quartiles of four independent experiments. Values of individual experiments are plotted on top of the boxes. Significance was assessed by repeated‐measures analysis of variance (ANOVA) with post hoc Tukey correction for multiple comparisons. Significant differences are marked by asterisks (*p < .05; **p < .01, ***p < .001). (D) Summary of the metabolic changes in NNMT_kd_ cells

To further investigate the consequences of NNMT knockdown on cellular (glutamine) metabolism, we performed a targeted metabolomics analysis, assessing intra‐ and extracellular levels of the different amino acids and TCA cycle intermediates (Table S2). In this setting, the accumulation of glutamine in NNMT_kd_ cells was confirmed (fc = 3.6, *p*_(Tukey)_ = .029). Interestingly, the levels of most other amino acids were also increased in NNMT_kd_ cells, together with the TCA cycle intermediates malate (fc = 1.5, *p*_(Tukey)_ = .007), fumarate (fc = 1.5, *p*_(Tukey)_ = .004) and α‐ketoglutarate (fc = 1.9, *p*_(Tukey)_ = .011) (Figure 3A). NNMT_kd_ cells secreted increased amounts of glutamine (fc = 1.4, *p*_(Tukey)_ = .021), glycine (fc = 3.2, *p*_(Tukey)_ = .025), alanine (fc = 1.2, *p*_(Tukey)_ = .012), α‐ketoglutarate (fc = 1.6, *p*_(Tukey)_ < .001) and citrate (fc = 1.5, *p*_(Tukey)_ = .002) (Figure 3B). Furthermore, NNMT_kd_ cells took up increased levels of aspartate (fc = 1.3, *p*_(Tukey)_ = .014) from the media and less pyruvate (fc = 0.7, *p*_(Tukey)_ = .045) and tyrosine (fc = 0.7, *p*_(Tukey)_ = .019) (Figure 3C). An overview of the metabolic changes in NNMT_kd_ cells is given in Figure 3D. We suspected the increased uptake of aspartate by NNMT_kd_ cells to be a mechanism to compensate for impaired glutamine metabolism in those cells, as aspartate is able to substitute for glutamine under glutamine‐deprived conditions.^49^ If this held true, cultivation in media with sufficient aspartate would rescue NNMT_kd_ cell viability. To test this hypothesis, we cultured NNMT_kd_ and control cells in glucose‐ and amino acid‐free media supplied with glutamine and/or aspartic acid. However, supplementation with aspartic acid did not rescue the viability of NNMT_kd_ cells (Figure S4A). Of note, the addition of glucose to the media had only a small benefit for cell viability when glutamine was present, showing the dependency of 786‐O cells on glutamine for cell survival, as described before^47^, ^50^ (Figure S4B). Importantly, glutamine accumulation in NNMT_kd_ cells did not lead to diminished levels of TCA cycle intermediates or amino acid synthesis, suggesting defects in other pathways that cause the observed impaired cell viability of NNMT_kd_ cells. In fact, accumulation of those metabolites could be a consequence of reduced viability, accompanied by a decreased demand for macromolecular synthesis.

### 3.4 Glutathione levels are unaffected in NNMT_kd_ cells, while levels of oxidised glutathione are diminished

**FIGURE 4:**
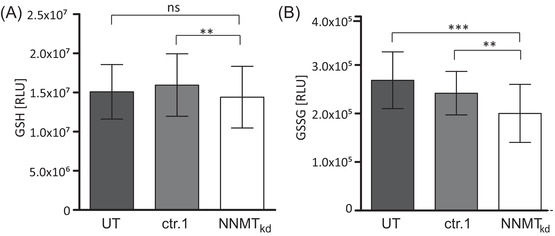
Glutathione measurements. (A) Glutathione (GSH) levels in cells transfected with control siRNA 1 (ctr.1) and nicotinamide‐N‐methyltransferase knockdown (NNMT_kd_) cells given as a percentage of levels in untreated cells (UT). (B) Percentage of oxidised GSH (GSSG) in ctr.1 and NNMT_kd_ cells compared to untreated cells. The bars represent the mean values +/‐ standard deviation of four independent experiments. Significance was assessed by repeated‐measures analysis of variance (ANOVA) with post hoc Tukey correction for multiple comparisons. Significance levels of differences are given in the graphs (ns: not significant; **p < .01; ***p < .001)

Glutamine is an important substrate for the synthesis of GSH (Figure 2C), which is needed to neutralise reactive oxygen species (ROS) that are generated by the electron transport chain of mitochondrial respiration. Especially in high‐grade ccRCC, the antioxidant response mediated by GSH becomes essential for tumour growth.^50^ We suspected that the reduced cell viability of NNMT_kd_ cells might be caused by impaired GSH‐mediated ROS neutralisation due to defective GSH synthesis in those cells. To assess this, we measured the levels of reduced GSH and the GSSG in NNMT_kd_ and control cells. Interestingly, GSH levels were unchanged in NNMT knockdown conditions (Figure 4A), whereas levels of GSSG were significantly decreased by 26.5% (*p*_(Tukey)_ = .001) and 17.2% (*p*_(Tukey)_ = .007) when compared to UT and ctr.1 control cells, respectively (Figure 4B). These results indicate that NNMT knockdown does not affect GSH synthesis. The observed decrease in GSSG levels might indicate a decreased demand for ROS neutralisation in NNMT_kd_ cells.

### 3.5 NNMT_kd_ cells have impaired mitochondrial function

**FIGURE 5:**
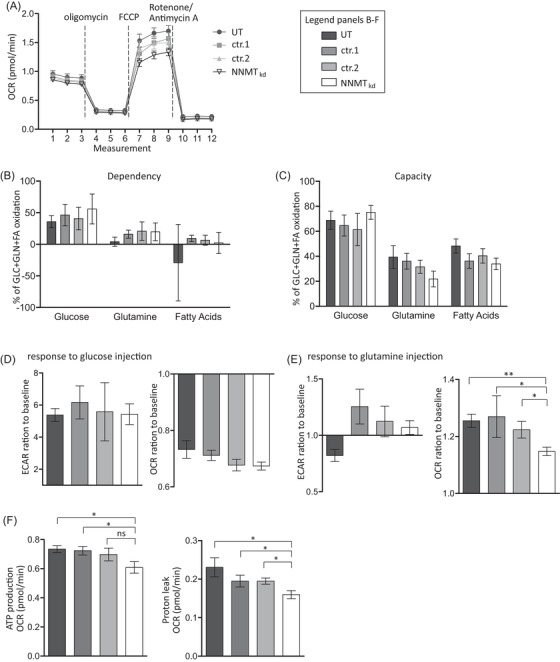
Extracellular flux analysis. (A) Oxygen consumption rate (OCR) of untreated 786‐O cells (UT), cells transfected with nontargeting siRNA controls 1 and 2 (ctr.1, ctr.2), and nicotinamide‐N‐methyltransferase knockdown (NNMT_kd_) cells. Compounds from the Mito‐Stress Test kit (Agilent) were used to modulate mitochondrial activity. The plotted values represent the means ± standard deviation (SD) of three independent experiments. (B) Dependency of NNMT_kd_ and control cells on glucose, glutamine and fatty acids (FA) as energy sources for mitochondrial respiration. The bars represent the mean values ± SD of three independent experiments. None of the differences reached statistical significance, as determined by Student's *t*‐test with Benjamini–Hochberg correction for multiple testing. (C) Capacity of NNMT_kd_ and control cells to use either glucose, glutamine, or FA if the remaining two fuel sources are inhibited. None of the differences reached statistical significance, as determined by Student's *t*‐test with Benjamini–Hochberg correction for multiple testing. (D) Changes in the extracellular acidification rate (ECAR) and OCR of NNMT_kd_ and control cells in response to an injection of 10 mM glucose (final concentration). The responses to glucose were measured 20 min after injection. The detected differences were not statistically significant. (E) Changes in ECAR and OCR in response to an injection of 2 mM glutamine (final concentration). Again, the responses were measured 20 min after injection. (F) Impact of NNMT_kd_ on the adenosine triphosphate (ATP) production rate and mitochondrial proton leakage. Significance levels, determined by Student's *t*‐test with Benjamini–Hochberg correction for multiple testing, are given in the plot (ns: not significant; *p < .05; **p < .01)

Since ROS are primarily produced during mitochondrial oxphos, diminished oxphos could explain the decreased levels of GSSG in NNMT_kd_ cells. To investigate the impact of NNMT knockdown on mitochondrial function, we performed extracellular flux analysis with mitochondrial modulators (Mito Stress Test, Agilent) in NNMT_kd_ and control cells. For comprehensiveness, we also looked at glycolytic function (Glyco Stress Test, Agilent), the second major cellular energy production pathway. NNMT knockdown did not affect glycolysis in 786‐O cells (Figure S5). In contrast, mitochondrial respiration was indeed impaired in NNMT_kd_ cells, especially under stressed conditions that are mimicked by the uncoupling agent trifluoromethoxy carbonylcyanide phenylhydrazone (FCCP) (Figure 5A). Consequently, maximal respiration (diff_(UT)_ = −0.31, *p*_(UT)_ = .019; diff_(ctr.1)_ = −0.22, *p*_(ctr.1)_ = .043; diff_(ctr.2)_ = −0.16, *p*_(ctr.2)_ = .060) and spare respiratory capacity (diff_(UT)_ = −0.26, *p*_(UT)_ = .009; diff_(ctr.1)_ = −0.18, *p*_(ctr.1)_ = .021; diff_(ctr.2)_ = −0.13, *p*_(ctr.2)_ = .030) were significantly diminished in NNMT_kd_ cells (Figure S6). The assay medium used contains many possible energy sources, including glucose, fatty acids, glutamine and other amino acids, as possible mitochondrial fuels. To assess which fuel sources are used in our setting, we performed the Mito Fuel Flex Test assay (Agilent). In this assay, cells’ dependencies on glucose, glutamine and long‐chain fatty acids are measured, as well as their capacity to use one of the fuel sources if the other two pathways are inhibited. 786‐O cells depend mainly on glucose for mitochondrial respiration, with no apparent differences between NNMT_kd_ and control cells (Figure 5B). However, the capacity of NNMT_kd_ cells to use glutamine as mitochondrial fuel is slightly diminished compared to UT (diff = 14.2%, *p* = .08) and ctr.1 (diff = 17.6%, *p* = .08) cells (Figure 5C). This is in line with our previous results that indicate defective glutamine metabolism in NNMT_kd_ cells. Defective glutamine shuttling into mitochondrial oxphos might also be the reason for the impaired viability of NNMT_kd_ cells. To further support this idea, we assessed the response of mitochondrial respiration and glycolysis in glucose‐ and glutamine‐deprived cells to an injection of either glucose or glutamine. When starved cells were provided with glucose, the extracellular acidification rate was increased approximately sixfold, implying enhanced glycolytic activity, whereas oxygen consumption, representing mitochondrial respiration, was reduced to approximately 0.7‐fold of baseline respiration (Figure 5D). There were no differences between NNMT_kd_ and control cells. The addition of glutamine resulted in only a minor increase in glycolysis in control and NNMT siRNA‐transfected cells and decreased glycolysis in untreated cells (Figure 5E, left panel). Mitochondrial respiration in control cells increased approximately 1.2‐fold (Figure 5E, right panel). Importantly, the increase in mitochondrial respiration in response to glutamine was diminished in NNMT_kd_ cells (diff_(UT)_ = −0.10, *p*_(UT)_ = .002; diff_(ctr.1)_ = −0.11, *p*_(ctr.1)_ = .047; diff_(ctr.2)_ = −0.07, *p*_(ctr.2)_ = .024). Furthermore, ATP production (diff_(UT)_ = −0.13, *p*_(UT)_ = .024; diff_(ctr.1)_ = −0.11, *p*_(ctr.1)_ = .024; diff_(ctr.2)_ = −0.09, *p*_(ctr.2)_ = .059) and proton leakage (diff_(UT)_ = −0.07, *p*_(UT)_ = .015; diff_(ctr.1)_ = −0.04, *p*_(ctr.1)_ = .030; diff_(ctr.2)_ = −0.04, *p*_(ctr.2)_ = .015) of cells supplied with glutamine was lower in NNMT_kd_ cells (Figure 5F), indicating decreased mitochondrial activity in those cells.

Next, to confirm the data of NNMT knockdown in 786‐O cells with regard to mitochondrial function, we performed analyses using the 2D ccRCC metastasis‐derived primary model RCC1. In line with the observation in 786‐O cells, mitochondrial function was diminished in NNMT_kd_ RCC1 cells, especially under stressed conditions (Figure S7). Again, maximal respiration (diff_(UT)_ = −0.38, *p*_(UT)_ = .048; diff_(ctr.1)_ = −0.29, *p*_(ctr.1)_ = .048; diff_(ctr.2)_ = −0.21, *p*_(ctr.2)_ = .048) and spare respiratory capacity (diff_(UT)_ = −0.32, *p*_(UT)_ = .020; diff_(ctr.1)_ = −0.20, *p*_(ctr.1)_ = .020; diff_(ctr.2)_ = −0.17, *p*_(ctr.2)_ = .027) were significantly decreased in NNMT_kd_ cells. Taken together, these experiments imply that NNMT supports the shuttling of glutamine into mitochondrial oxphos, which increases cell respiration and viability.

### 3.6 Glycolysis, oxidative phosphorylation and apoptosis pathways are upregulated in NNMT_kd_ cells

**FIGURE 6:**
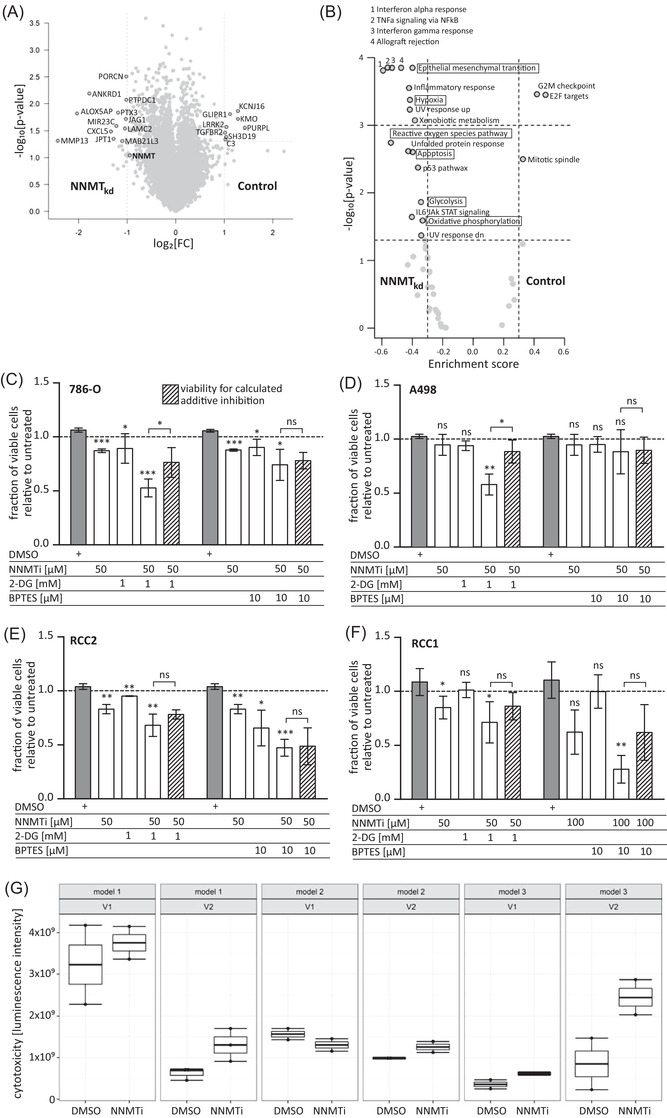
(A) Differential gene expression in nicotinamide‐N‐methyltransferase knockdown (NNMT_kd_) and control siRNA 1‐transfected cells (control). *p*‐Values were calculated by Student's *t*‐test. (B) Gene set enrichment analysis (GSEA) in NNMT_kd_ and ctr.1 cells (control) with the Hallmark gene set of the molecular signatures database (MSigDB). Enrichment scores and *p*‐values were calculated with the piano package (see Section 2). (C–F) Cell viability of the clear cell renal cell carcinoma (ccRCC) cell lines 786‐O and A498, the ccRCC tumour‐derived primary model RCC2, and the ccRCC metastasis‐derived primary model RCC1 treated with an inhibitor of nicotinamide‐N‐methyltransferase (NNMTi) alone or in combination with 2‐deoxy‐D‐glucose (2‐DG) or BPTES for 24 h. The shaded bars represent the additive inhibitory effects calculated by summing the inhibitory effects of the individual inhibitors. Bars represent the mean values ± standard deviation of three to five independent experiments. Significance was determined by Student's *t*‐test with Benjamini–Hochberg correction for multiple comparisons. Significance levels are indicated in the graphs (ns: not significant; *p < .05; **p < .01; ***p < .001). (G) Cell cytotoxicity in three different 3D air–liquid interface (ALI) models of ccRCC treated with 100 μM NNMTi or DMSO (1:1000) as a control for 24 h. For each model, two independent experiments were performed in duplicate or triplicate wells. Individual fluorescence measurements, indicative of cell cytotoxicity, are plotted on top of box plots

To further analyse the effect of NNMT knockdown on gene expression levels, we next compared whole transcriptome gene expression of NNMT_kd_ in 786‐O cells versus control cells (ctr.1). NNMT is known as a phase II metabolic enzyme that methylates its substrates by the transfer of a methyl group from SAM. It has been proposed that high NNMT expression leads to the depletion of cellular SAM levels, generating a methylation sink in those cells, leading to altered epigenetic patterns and gene expression.^12^ In general, we observed more downregulated genes in NNMT_kd_ cells than in control cells (Figure 6A). Notably, genes involved in glutamine metabolism, such as GLS and glutamate‐ammonia ligase, or genes responsible for glutamine uptake were not differentially regulated in NNMT_kd_ cells (Table S3). Next, we used GSEA to identify deregulated pathways in NNMT_kd_ cells (Figure 6B). Negative enrichment scores (es) in the plot indicate enrichment in NNMT_kd_ cells, while positive scores indicate enrichment in control cells. Interestingly, the ‘reactive oxygen species pathway’ (es = −0.54, *p* = 1.8 × 10^–3^), ‘apoptosis’ (es = −0.40, *p* = 2.5 × 10^–3^), ‘glycolysis’ (es = −0.34, *p* = 1.4 × 10^–2^) and ‘oxidative phosphorylation’ (es = −0.33, *p* = 2.6 × 10^–2^) were upregulated in NNMT_kd_ cells, whereas ‘G2M checkpoint’ (es = 0.42, *p* = 3.5 × 10^–4^), ‘E2F targets’ (es = 0.47, *p* = 3.5 × 10^–4^) and ‘mitotic spindle’ (es = 0.33, *p* = 3.2 × 10^–3^) were downregulated. Downregulation of ‘G2M checkpoint’, ‘E2F targets’ and ‘mitotic spindle’ genes could indicate decreased replication of NNMT_kd_ cells and, along with upregulation of ‘apoptosis’ genes, could explain the decreased viability of NNMT_kd_ that we observed in our experiments. Upregulation of ‘glycolysis’ and ‘oxidative phosphorylation’ genes could be an attempt of NNMT_kd_ cells to compensate for the decreased mitochondrial function that is caused by defective glutamine metabolism in those cells. Other deregulated pathways include ‘epithelial mesenchymal transition’ (es = −0.40, *p* = 1.4 × 10^–4^), ‘xenobiotic metabolism’ (es = −0.38, *p* = 8.4 × 10^–4^) and ‘hypoxia’ (es = −0.42, *p* = 4.2 × 10^–4^). Whether deregulation is a consequence of an altered methylation potential in NNMT_kd_ cells or rather a consequence of impaired cellular function and fitness, or a combination of both, cannot be answered in our study.

### 3.7 Investigation of NNMT inhibition in ccRCC cell lines, primary cultures and ex vivo 3D models

From the in vitro data, we speculated that by inducing the expression of genes involved in glycolysis and oxphos, NNMT_kd_ cells try to meet their energy demand in the face of impaired glutamine utilisation in mitochondrial oxphos. In this stressed scenario, NNMT‐depleted cells might be more vulnerable to other therapies that target, for example, glycolysis, representing the other major energy production pathway. To test this hypothesis, we combined the NNMT inhibitor NNMTi^23^ with the glucose analogue 2‐DG, a potent inhibitor of glycolysis, or BPTES, an inhibitor of GLS, and monitored cell viability of 786‐O and A498 cell lines, and primary ccRCC models (RCC1 and RCC2). The RCC models represent a primary tumour‐derived ccRCC model and a model derived from a ccRCC lymph node metastasis, respectively. All models expressed NNMT (Figure S8A). The specificity of the NNMT inhibitor was verified by measuring the NNMT‐dependent metabolite 1‐MNA in inhibitor‐treated and control 786‐O cells (Figure S9).

Treatment of all tested models with 50 or 100 μM NNMTi alone significantly reduced cell viability compared to DMSO‐treated control cells (786‐O: fc_50μM_ = 0.82, *p*_50μM_ = 5.29 × 10^–7^; RCC2: fc_50μM_ = 0.80, *p*_50μM_ = .005; RCC1: fc_50μM_ = 0.78, *p*_50μM_ = .012) (Figures 6C–F and S10). A498 cells were the least sensitive, and only the maximal tested dose of 100 μM NNMTi led to a significant inhibition of cell viability (fc_100μM_ = 0.49, *p*_100μM_ = .021) (Figure S10). In all tested models, the inhibitory effect of 50 μM NNMTi could be enhanced when combined with 1 mM 2‐DG (786‐O: fc_(NNMTi+2‐DG)_ = 0.50, *p*_(NNMTi+2‐DG)_ = 9.16 × 10^–7^; A498: fc_(NNMTi+2‐DG)_ = 0.57, *p*_(NNMTi+2‐DG)_ = .004; RCC2: fc_(NNMTi+2‐DG)_ = 0.66, *p*_(NNMTi+2‐DG)_ = .004; RCC1: fc_(NNMTi+2‐DG)_ = 0.66, *p*_(NNMTi+2‐DG)_ = .012). The combination of NNMTi with 2‐DG in 786‐O and A498 even had a synergistic inhibitory effect, demonstrated by a significantly stronger inhibition of the compound combination than the calculated additive inhibition of the single treatments (shaded bars in the plots: *p*_(786‐O)_ = .011, *p*_(A498)_ = .022). Combination with 10 μM BPTES enhanced the inhibitory effect of NNMTi only in 786‐O (fc_(NNMTi+BPTES)_ = 0.70, *p*_(NNMTi+BPTES)_ = .025), RCC2 (fc_(NNMTi+BPTES)_ = 0.46, *p*_(NNMTi+BPTES)_ = .001) and RCC1 (fc_(NNMTi+BPTES)_ = 0.25, *p*_(NNMTi+BPTES)_ = .007) cells but not in A498. No synergism of the combined NNMTi/BPTES treatment was observed. These data show that NNMT inhibition could provide an additional option for ccRCC therapy in metastatic patients and might render cells more vulnerable to other therapeutic interventions.

Notably, NNMT inhibition might not only inhibit cancer cells themselves but also impact the activation status of tumour‐infiltrating T cells through its reaction product 1‐MNA.^51^ Along this line, the first data indicate that 1‐MNA treatment of pre‐stimulated T cells from healthy volunteers increases surface expression of the immune checkpoint PD1 on CD4+ and CD8+ T‐cell populations (Figure S11).

To extend the findings of NNMT inhibition on cancer cells from 2D to more physiological 3D tumour models, we used patient‐derived ex vivo ALI models of three different ccRCC tumours.^22^ Inhibitor‐induced cytotoxicity was measured after treatment of the ALI models with 100 μM NNMTi for 24 h. We observed induced cytotoxicity of NNMTi‐treated ALIs in two of the three tested models (Figure 6G). All models expressed NNMT, although at varying levels (Figure S8B).

## 4 DISCUSSION

With this study, we aimed to better understand the cellular function of NNMT in ccRCC and to elucidate its role as a potential target for therapy of metastatic RCC disease. NNMT has been shown to be highly expressed in several cancer entities, including ccRCC,^1^, ^2^ and expression was associated with more aggressive tumours and worse outcome in most studies (reviewed in Ref.^3^).^52^ In agreement, expression of NNMT was high in our patient cohort of primary ccRCC and the TCGA KIRC cohort, and mRNA expression correlated significantly with inferior patient survival. NNMT protein expression in cohort 1 showed the same trend of worse survival of patients with high NNMT‐expressing tumours. In addition to primary tumour data, we report for the first time high expression of NNMT in ccRCC‐derived metastases irrespective of the organ. The function NNMT may play in tumourigenesis is still a matter of discussion. Ulanovskaya et al.^12^ proposed that by consuming SAM, NNMT generates a methylation sink in cells, which leads to altered histone methylation and expression of oncogenes. While no effect on DNA methylation was observed in this study, Jung et al.^13^ reported increased DNA methylation and differentiation in NNMT‐depleted glioblastoma stem cells. In another study, NNMT was reported to influence the methylation of tumour suppressors and oncogenes directly, thereby supporting cancer cell survival.^53^ NNMT was also shown to regulate histone methylation in cancer‐associated fibroblasts, supporting oncogenic remodelling of the metastasis‐associated stroma. We did not observe prominent stromal NNMT expression in tissue sections of either primary ccRCC or ccRCC‐derived metastasis (Figure S12), indicating that, in ccRCC, tumour cell expression of NNMT plays the dominant role. This observation is supported by single‐cell gene expressing data, showing the highest NNMT expression levels in the ccRCC tumour compartment. Eckert et al. and several other studies identified NNMT as a metastasis‐associated gene,^54^, ^55^, ^56^, ^57^ possibly acting by inducing the expression of matrix‐metalloproteinase 2 (MMP‐2)^8^ and maintaining dedifferentiated mesenchymal‐like gene expression.^13^, ^58^ In agreement, we observed deregulated metastasis‐associated genes in NNMT_kd_ knockdown cells in our study, including MMP‐13, laminin subunit gamma 2 (LAMC2) and alpha‐1,6‐mannosylglycoprotein 6‐beta‐N‐acetylglucosaminyltransferase (MGAT5). NNMT has also been shown to regulate autophagy in liver and breast cancer cells^59^, ^60^ and to confer resistance to radiation and drug therapy.^53^, ^61^, ^62^, ^63^ In our cohort of ccRCC‐derived metastases, we observed slightly lower expression of NNMT in TKI‐pretreated tissues, which may be relevant for the therapeutic application of NNMT. However, this observation is based on the analysis of very few samples and needs further investigation.

In the present study, we show that NNMT knockdown impairs mitochondrial respiration and reduces the viability of RCC cells. We propose that the effect is caused by a defect in the shunting of glutamine through the TCA cycle to feed mitochondrial oxphos in NNMT_kd_ cells. VHL‐deficient RCC cells depend on extracellular glutamine for lipid synthesis, as highly active aerobic glycolysis prevents glucose from fueling the TCA cycle in these cells.^47^, ^64^ In addition, glutamine is used to produce GSH, which becomes increasingly important in advanced ccRCC to combat the accumulation of ROS in actively proliferating tumour cells.^17^, ^50^, ^65^, ^66^, ^67^ In our study, glutamine accumulation in NNMT_kd_ cells did not affect the levels of reduced GSH. In contrast, we observed decreased levels of GSSG, which is in line with reduced oxidative stress due to impaired mitochondrial respiration in these cells. Importantly, in contrast to other recently published studies,^58^, ^62^, ^68^ we did not observe an impact of NNMT knockdown on glycolytic function, the expression of individual glycolysis‐related genes, or sensitivity to glucose deprivation. We also did not observe altered expression of individual genes involved in glutamine metabolism in NNMT_kd_ cells. On the pathway level, however, NNMT_kd_ cells upregulate glycolysis, oxphos and ROS pathways, possibly in an attempt to compensate for the defect in mitochondrial respiration and cellular bioenergetics.

Taken together, we propose that NNMT supports oxphos in ccRCC tumours by regulating the shuttling of glutamine through the TCA cycle. Together with the utilisation of glutamine for GSH production and ROS neutralisation, this might underlie the glutamine avidity of ccRCC, providing cancer cells with sufficient energy for proliferation. It was recently proposed that, beyond ATP production, mitochondrial respiration is essential for cell proliferation by maintaining cytoplasmic aspartate levels.^69^, ^70^, ^71^ In our study, we observed increased uptake of aspartate by NNMT_kd_ cells, possibly as a consequence of impaired mitochondrial function. However, supplementing NNMT_kd_ cells with aspartic acid could not rescue cell viability under glutamine‐deprived conditions in our study. In addition, intracellular levels of aspartate in NNMT_kd_ cells were higher than those in controls, leading us to the conclusion that aspartate deficiency does not seem to underlie the impaired viability of NNMT_kd_ cells. In contrast, we observed impaired ATP production in NNMT_kd_ cells. Hence, we speculated that inhibition of NNMT would render cells more vulnerable to glycolysis inhibition by 2‐DG, since the combination of inhibitors would affect both energy production pathways. Indeed, dual inhibition of NNMT (using the inhibitor NNMTi^23^) and glycolysis in both ccRCC cell lines (786‐O and A498) and two 2D ccRCC primary models derived from tumour (RCC2) and metastasis (RCC1) led to strongly decreased cell viability. Whether the action of sirtuin proteins is involved in the regulation of NNMT‐mediated glutamine shuttling by the induction of mitochondrial complex I activity and ATP synthesis, as suggested by Liu et al.^10^ and Parsons et al.^11^ in neuroblastoma, needs further experimental evaluation. We indeed observed slightly reduced sirtuin 1 (SIRT1) and growth arrest and DNA damage inducible alpha (GASS45A) expression in NNMT_kd_ cells, although other SIRT1‐regulated genes were not affected (data not shown).

In our study, we used the primary ccRCC‐derived VHL‐deficient 786‐O cell line for mechanistic investigations, which is an accepted ccRCC in vitro model that is able to form ccRCC tumours in nude mice and maintain the characteristics of ccRCC tumours, such as vimentin and CD10 surface expression and secretion of high levels of vascular endothelial growth factor (VEGF).^72^, ^73^ Metabolically 786‐O cells also resemble ccRCC tumours in terms of Warburg effect aerobic glycolysis, fatty acid and glutamine metabolism.^50^ We further confirmed the influence of NNMT depletion on mitochondrial respiration in a ccRCC metastasis‐derived primary cell culture (RCC1).

Notably, the effect of the NNMT inhibitor NNMTi was investigated in two ccRCC cell lines (786‐O and A498), two patient‐derived 2D models (RCC1 and RCC2) and three ex vivo patient‐derived 3D ALI ccRCC models. It has already been demonstrated^22^, ^74^ that ALI models preserve the complex architecture of ccRCC, including even tumour infiltrating lymphocytes (TILs). Moreover, ex vivo tumour models have been shown to well recapitulate drug responses in patients^75^, ^76^ and are therefore a powerful system to study new drugs and drug combinations. NNMTi treatment‐induced cytotoxicity was confirmed in two out of three patient‐derived ccRCC ALI models, strongly corroborating the therapeutic potential of NNMT inhibition in vivo.

Thus, we propose that due to the broad expression of NNMT in primary ccRCC tumours and metastases, its association with patient survival, and its molecular functions, NNMT is a promising drug target in ccRCC. Small‐molecule inhibitors of NNMT have been developed in recent years and are discussed as therapeutics for metabolic diseases, such as diabetes, obesity and fatty liver disease.^77^ Preclinical studies have demonstrated beneficial effects of NNMT inhibition in obese mice.^78^, ^79^, ^80^ Regarding NNMT inhibition in cancer, one study showed decreased tumour burden and cancer cell proliferation in an orthotropic mouse model of ovarian cancer treated with an NNMT inhibitor.^54^ In addition, a newly published study demonstrated the tumour‐promoting and immune‐suppressing effects of 1‐MNA, the NNMT‐dependent metabolite of nicotinamide, in ovarian cancer.^51^ Kilgour et al. showed that 1‐MNA secreted by NNMT‐expressing fibroblasts and cancer cells is taken up by T cells in the tumour microenvironment. In response, T cells secrete increased tumour necrosis factor‐α and decreased interferon‐γ, resulting in the promotion of tumour growth and reduced cytotoxicity. In agreement with the immune suppressive function, we observed a correlation of NNMT expression with the number of regulatory T cells in the tumour tissue of cohort 1 (Figure S13). Furthermore, the first data show that 1‐MNA, alone and in combination with TGFβ, increases the amount of PD1‐expressing CD4 T cells and, to a lesser extent, CD8 T cells. Although these data need further experimental evaluation, it seems likely that, similar to ovarian cancer, 1‐MNA acts as an immune‐suppressive metabolite in ccRCC, suggesting a dual effect of NNMT inhibition in ccRCC. As mentioned before, NNMT is highly expressed in the liver but also in normal kidneys and other organs, and therefore, the safety of NNMT inhibition in humans needs careful evaluation. The first data in animals did not report observable adverse effects of NNMT inhibition or NNMT knockdown.^79^, ^80^, ^81^ In addition to molecular targeting, NNMT‐derived peptides that are specifically presented by HLA molecules on ccRCC tumours could allow immunologic targeting of NNMT‐expressing primary tumours and metastases.^14^

## 5 CONCLUSIONS

Our study shows that NNMT is an important regulator of glutamine metabolism in ccRCC, with consequences for mitochondrial function and cellular fitness. NNMT inhibition impairs ccRCC metabolism alone or in combination with other agents and drives primary and metastatic cancer cells into cell death. Despite a growing repertoire of treatment options, advanced ccRCC is still not curable. To date, highly specific NNMT inhibitors have been developed without obvious safety issues in mice. Therefore, beyond its currently discussed role as a drug target in metabolic conditions, NNMT represents a promising new target for the treatment of ccRCC and potentially other cancer entities.^82^

## CONFLICT OF INTEREST

Jens Bedke: personal honoraria for speaker, consultancy or advisory role—AstraZeneca, Astellas, BMS, Eisai, Ipsen, MSD, Novartis, Roche, EUSA Pharma, Nektar and Pfizer; institutional financial interests which has been paid directly to your institution—Eisai, Ipsen, MSD, Novartis, Roche and Pfizer. Arnulf Stenzl: consultancies, honoraria or study participation from Bayer, BMS, Immatics, Novartis, Pfizer and Roche. Steffen Rausch: honoraria for speaker, advisory role—Astellas, Bayer, Pfizer and Merck. The remaining authors have nothing to disclose.

## Supporting information

Supporting Information (Additional file 1.pdf)
