# Supplementary material for: Nicotinamide‐N‐methyltransferase is a promising metabolic drug target for primary and metastatic clear cell renal cell carcinoma
Source: Clin Transl Med. 2022 Jun 8;12(6):e883. doi: 10.1002/ctm2.883 (PMC9178377; doi:10.1002/ctm2.883)
Supplement: Supplementary file 1 — Supporting Information (Additional file 1.pdf) [file CTM2-12-e883-s001.pdf]

Additional File 1: Supplementary Tables and Figures

To:

Anna Reustle[1], Lena-Sophie Menig[1], Patrick Leuthold[1], Ute Hofmann[1], Viktoria Stühler[2], Christian Schmees[3], Michael Becker[4], Mathias Haag[1], Verena Klumpp[1], Stefan Winter[1], Florian A. Büttner[1], Steffen Rausch[2], Jörg Hennenlotter[2], Falko Fend[5], Marcus Scharpf[5], Arnulf Stenzl[2], Jens Bedke[2,6], Matthias Schwab[1,6,7,8*†], Elke Schaeffeler[1,8†]

Nicotinamide-N-methyltransferase is a promising metabolic drug target for primary and metastatic clear cell renal cell carcinoma

Supplementary Tables:

Table S1: Deregulated features in $NNMT_{kd}$ cells detected by non-targeted metabolomics analysis.
Table S2: Levels of amino acids and TCA intermediates in $NNMT_{kd}$ and control cells.
Table S3: Expression of genes involved in glutamine metabolism in $NNMT_{kd}$ and control cells.

Supplementary Figures:

Figure S1: NNMT antibody validation and knockdown efficiency.
Figure S2: NNMT expression and clinical parameters.
Figure S3: NNMT expression in patients with multiple metastases.
Figure S4: Aspartate and glucose dependency.
Figure S5: Glycolysis stress test and glycolytic parameters of 786-O cells.
Figure S6: Mitochondrial stress test and mitochondrial parameters in 786-O cells.
Figure S7: Mitochondrial stress test and mitochondrial parameters in the ccRCC metastasis derived cell culture RCC1.
Figure S8: NNMT expression in cell culture models used in the study.
Figure S9: Validation of NNMTi inhibitory activity.
Figure S10: Concentration dependent inhibition of cell viability by NNMTi, 2-DG and BPTES.
Figure S11: The impact of the metabolite 1-MNA on PD1 expression of pre-activated T cells.
Figure S12: Immunohistochemistry of primary ccRCC tissue, paired non-tumor tissue and ccRCC-derived metastases.
Figure S13: NNMT expression and regulatory T cells in tumor tissue of cohort 1.

Table S1: Deregulated features in $NNMT_{kd}$ cells detected by non-targeted metabolomics analysis. Only features that were differentially regulated (FC>1.2 for upregulated in $NNMT_{kd}$ and FC <0.83 for downregulated in $NNMT_{kd}$, p-value<0.05) in $NNMT_{kd}$ cells compared to all included controls (ctr.1, ctr.2 and UT) are shown in the table. Fold-changes that remained significant (p<0.05) after Benjamini-Hochberg correction for multiple testing (n_{neg}=527, n_{pos}=526) are printed in bold. IM indicates the ionization mode. The Level of Assignment (LoA) was determines as described [1] and is given in the table. A LoA of 1 indicates that the accurate mass is detected in the Metlin online data base [2]. A LoA of 2 indicates a match between the MS/MS spectra of the detected feature and the dedicated metabolite. A LoA of 3 indicates that the metabolite could be assigned by the accurate mass and retention time (RT) matching. For most features, no metabolite could be assigned with sufficient certainty.

						**ctr.1**		**ctr.2**		**UT**	
**IM**	**Mass**	**RT**	**Formula**	**Metabolite**	**LoA**	**FC**	**p-value**	**FC**	**p-value**	**FC**	**p-value**
neg	146.0687	10.51	C5H10N2O3	Glutamine	3	4.51	3.25E-04	4.30	8.07E-03	4.73	3.77E-04
neg	102.0684	5.25	C5H10O2	Pentanoate	1	0.38	1.10E-03	0.51	1.50E-02	0.39	7.21E-03
neg	179.0253	5.97				3.29	6.31E-03	2.61	1.01E-02	2.11	2.64E-02
neg	145.1104	5.25	C7H15NO2	Acetylcholine	1	0.41	4.89E-03	0.51	1.24E-03	0.38	1.50E-02
neg	320.0341	10.51				3.78	6.68E-04	3.95	2.13E-03	5.73	9.88E-05
neg	189.1002	5.25	C8H15NO4	Castanospermine	1	0.47	2.33E-03	0.55	1.59E-02	0.45	2.56E-02
neg	359.155	5.25				0.50	3.61E-03	0.64	1.31E-02	0.51	4.07E-03
neg	259.0812	6.48	C9H13N3O6	N-hydroxycytidine	1	0.41	2.58E-02	0.30	9.75E-04	0.58	2.13E-02
pos	245.1627	5.24	C12H23NO4	2-Methylbutyroylcarnitine	2	**0.38**	**1.42E-03**	1.00	8.21E-03	0.39	7.89E-03
pos	136.0634	5.91	C7H8N2O	Trigonellinamide	2	**0.77**	**6.92E-04**	**1.00**	**8.20E-05**	0.82	2.31E-02
pos	103.0999	4.02				**1.61**	**6.69E-03**	1.00	4.39E-02	1.28	2.54E-02
pos	231.147	5.96	C11H21NO4	Butyryl-L-carnitine	2	0.62	1.26E-02	1.01	1.46E-02	0.52	1.01E-02
pos	128.0835	2.14	C7H12O2			**3.89**	**4.78E-04**	1.00	2.97E-03	2.70	2.09E-03
pos	129.0426	10.48	C5H7NO3			**3.51**	**2.27E-05**	**1.00**	**2.20E-05**	**5.17**	**5.50E-04**
pos	743.5475	2.24				2.63	2.70E-02	1.02	4.82E-02	2.71	1.39E-02
pos	743.5456	2.33				1.54	1.58E-02	1.01	4.97E-02	1.87	3.30E-03
pos	561.8808	6.54				**1.43**	**5.46E-05**	1.00	3.27E-03	1.23	8.32E-03
pos	789.53	2.22				2.34	2.36E-02	1.02	2.27E-02	2.27	1.67E-02
pos	771.5776	2.23				1.66	4.25E-02	1.03	4.35E-02	1.65	1.61E-02
pos	354.9258	19.1				**1.40**	**6.10E-03**	1.00	2.12E-02	1.24	2.08E-02
pos	89.0475	9.52				1.30	1.91E-02	1.01	8.15E-03	1.43	6.69E-04
pos	775.5504	2.07				1.96	1.94E-02	1.01	4.52E-02	1.71	1.59E-02
pos	227.0901	4.95				3.13	4.49E-02	1.03	3.52E-02	5.31	2.57E-02
pos	336.1349	6.54				**1.33**	**3.29E-05**	**1.00**	**3.75E-04**	1.21	3.83E-03
pos	111.0432	4.94				3.21	4.75E-02	1.03	2.65E-02	5.51	2.82E-02
pos	765.5302	2.24				2.51	1.45E-02	1.01	2.74E-02	2.57	6.99E-03
pos	745.5588	2.33				1.56	2.24E-02	1.02	2.94E-02	1.58	1.17E-02
pos	481.3528	5.04	C24H52NO6P	PC(O-16:0/0:0)	1	**0.39**	**4.93E-03**	**1.00**	**6.15E-04**	0.40	4.43E-03
pos	509.384	4.78	C26H56NO6P	PC(O-18:0/0:0)	1	0.35	1.54E-02	1.01	4.17E-03	0.42	3.23E-02
pos	323.1398	5.24	C19H19N2O3			**0.53**	**4.98E-03**	1.00	1.01E-02	0.50	8.29E-03

pos	186.0887	5.24	C9H14O4			**0.50**	**3.48E-04**	1.00	3.38E-02	0.53	5.68E-03
pos	400.9296	5.9				**0.70**	**5.49E-03**	1.00	8.29E-03	0.76	1.08E-02
pos	137.0477	7.71	C7H7NO2	Trigonelline	3	**0.63**	**5.07E-03**	1.00	4.14E-03	0.73	4.39E-03
pos	272.1272	5.91				**0.27**	**9.22E-04**	**1.00**	**4.14E-04**	0.41	2.14E-02
pos	664.8286	19.1				**1.39**	**4.20E-03**	1.00	1.59E-02	1.29	4.17E-03

Table S2: Levels of amino acids and TCA intermediates in $NNMT_{kd}$ and control cells. The metabolites were measured by a targeted metabolomics approach, as detailed in Materials and Methods. The values represent mean (4 replicates) absolute levels normalized by cell number (pmol/3.3x10^5 cells). Negative extracellular levels indicate that the metabolites are taken up by cells, positive extracellular levels represent secretion.

	**Intracellular**			**Extracellular**		
**Metabolite**	**UT**	**ctr.1**	**NNMT**	**UT**	**ctr.1**	**NNMT**
a-Ketoglutarate	61.20	58.95	89.86	1.04	0.85	1.38
Alanine	876.37	761.76	1325.93	291.32	373.61	456.64
Arginine	57.80	49.02	77.77	-8.42	-9.59	-6.01
Aspartate	2223.86	1974.93	3581.99	-12.39	-10.50	-14.21
Citrate	73.87	83.84	101.57	1.41	1.16	1.72
Citrulline LC-MS	na	na	na	0.16	0.15	0.18
Fumarate	143.49	130.75	194.69	0.04	0.01	0.01
Glutamate	13389.32	12541.89	21224.09	48.06	56.93	51.72
Glutamine	21.27	22.37	79.00	125.06	203.68	283.26
Glycine	2055.23	1702.75	2482.25	5.81	3.86	8.06
Histidine	44.52	48.75	55.75	4.10	3.87	4.56
Hydroxyglutaric acid	32.26	35.75	37.37	0.08	0.16	0.21
Isoleucine	126.98	111.33	151.80	-38.29	-34.69	-36.36
Leucine	195.08	170.36	231.08	-52.85	-50.37	-52.12
Lysine	72.81	62.30	93.84	-18.06	-21.57	-16.89
Malate	211.15	209.79	316.27	0.67	0.66	0.81
Methionine	33.72	30.68	43.91	-4.23	-4.73	-3.88
Ornithine LC-MS	na	na	na	3.44	3.42	3.79
Phenylalanine	117.57	96.59	138.25	-8.16	-8.72	-8.12
Proline	988.14	977.69	1545.05	8.23	7.22	9.45
Pyruvate	327.44	271.87	217.91	-38.64	-44.01	-32.51
Serine	58.20	53.83	66.46	-36.06	-36.25	-33.98
Threonine	328.90	250.20	396.49	-18.01	-17.82	-17.74
Tryptophan	26.94	22.51	32.32	-1.53	-1.71	-1.40
Tyrosine	92.84	77.12	112.58	-4.08	-5.02	-3.78
Valine	170.27	142.21	194.73	-28.30	-27.48	-27.34

Table S3: Expression of genes involved in glutamine metabolism in NNMTkd and control cells. Genes were selected based on their participation in glutamine-related pathways included in the Molecular Signatures Database (MSigDB) [3]. Differential expression in $NNMT_{kd}$ and ctr.1 is shown in the table.

ID	Symbol	Signatures	$NNMT_{kd}$		Ctr.1		p-value	fc
			mean	SD	mean	SD		
51166	AADAT	GO_GLUTAMATE_METABOLIC_PROCESS, GO_GLUTAMINE_FAMILY_AMINO_ACID_METABOLIC_PROCESS	5.39	0.08	5.33	0.02	0.52	0.053
122622	ADSSL1	GO_GLUTAMINE_FAMILY_AMINO_ACID_METABOLIC_PROCESS, GO_GLUTAMINE_METABOLIC_PROCESS	6.03	0.00	5.99	0.12	0.68	0.048
79814	AGMAT	GO_GLUTAMINE_FAMILY_AMINO_ACID_METABOLIC_PROCESS	3.59	0.09	3.53	0.40	0.86	0.064
5832	ALDH18A1	GLUTAMINE_FAMILY_AMINO_ACID_METABOLIC_PROCESS, GO_GLUTAMATE_METABOLIC_PROCESS, GO_GLUTAMINE_FAMILY_AMINO_ACID_BIOSYNTHETIC_PROCESS, GO_GLUTAMINE_FAMILY_AMINO_ACID_METABOLIC_PROCESS	8.57	0.06	8.83	0.14	0.20	-0.269
8659	ALDH4A1	GLUTAMINE_FAMILY_AMINO_ACID_METABOLIC_PROCESS, GO_GLUTAMATE_METABOLIC_PROCESS, GO_GLUTAMINE_FAMILY_AMINO_ACID_BIOSYNTHETIC_PROCESS, GO_GLUTAMINE_FAMILY_AMINO_ACID_CATABOLIC_PROCESS, GO_GLUTAMINE_FAMILY_AMINO_ACID_METABOLIC_PROCESS	5.95	0.04	5.91	0.08	0.59	0.043
7915	ALDH5A1	GO_GLUTAMATE_METABOLIC_PROCESS, GO_GLUTAMINE_FAMILY_AMINO_ACID_METABOLIC_PROCESS, GO_GLUTAMINE_METABOLIC_PROCESS, GO_GLUTATHIONE_METABOLIC_PROCESS	5.07	0.03	5.08	0.12	0.95	-0.006
144193	AMDHD1	GO_GLUTAMATE_METABOLIC_PROCESS, GO_GLUTAMINE_FAMILY_AMINO_ACID_METABOLIC_PROCESS	4.51	0.01	4.47	0.12	0.72	0.039
383	ARG1	GLUTAMINE_FAMILY_AMINO_ACID_METABOLIC_PROCESS, GO_GLUTAMINE_FAMILY_AMINO_ACID_CATABOLIC_PROCESS, GO_GLUTAMINE_FAMILY_AMINO_ACID_METABOLIC_PROCESS	3.09	0.03	3.02	0.22	0.73	0.072
384	ARG2	GO_GLUTAMINE_FAMILY_AMINO_ACID_METABOLIC_PROCESS	7.28	0.08	8.02	0.11	0.02	-0.737
420	ART4	GO_GLUTAMINE_FAMILY_AMINO_ACID_METABOLIC_PROCESS, GO_GLUTAMINE_METABOLIC_PROCESS	2.61	0.10	2.59	0.13	0.88	0.019
435	ASL	GLUTAMINE_FAMILY_AMINO_ACID_METABOLIC_PROCESS, GO_GLUTAMINE_FAMILY_AMINO_ACID_BIOSYNTHETIC_PROCESS, GO_GLUTAMINE_FAMILY_AMINO_ACID_CATABOLIC_PROCESS, GO_GLUTAMINE_FAMILY_AMINO_ACID_METABOLIC_PROCESS	6.73	0.04	6.63	0.13	0.47	0.099
440	ASNS	GO_GLUTAMINE_FAMILY_AMINO_ACID_METABOLIC_PROCESS, GO_GLUTAMINE_METABOLIC_PROCESS	8.36	0.03	8.42	0.09	0.57	-0.052

54529	ASNSD1	GO_GLUTAMINE_FAMILY_AMINO_ACID_METABOLIC_PROCESS, GO_GLUTAMINE_METABOLIC_PROCESS	6.60	0.07	6.71	0.23	0.62	-0.109
80150	ASRGL1	GLUTAMINE_FAMILY_AMINO_ACID_METABOLIC_PROCESS, GO_GLUTAMINE_FAMILY_AMINO_ACID_CATABOLIC_PROCESS, GO_GLUTAMINE_FAMILY_AMINO_ACID_METABOLIC_PROCESS	5.15	0.02	4.99	0.10	0.25	0.165
445	ASS1	GO_GLUTAMINE_FAMILY_AMINO_ACID_BIOSYNTHETIC_PROCESS, GO_GLUTAMINE_FAMILY_AMINO_ACID_METABOLIC_PROCESS	7.28	0.04	7.34	0.06	0.37	-0.064
790	CAD	GO_GLUTAMINE_FAMILY_AMINO_ACID_BIOSYNTHETIC_PROCESS, GO_GLUTAMINE_FAMILY_AMINO_ACID_METABOLIC_PROCESS, GO_GLUTAMINE_METABOLIC_PROCESS, GO_GLUTATHIONE_METABOLIC_PROCESS	6.70	0.01	6.74	0.01	0.08	-0.046
79094	CHAC1	GO_GLUTATHIONE_METABOLIC_PROCESS	7.95	0.07	8.18	0.09	0.12	-0.232
494143	CHAC2	GO_GLUTATHIONE_METABOLIC_PROCESS	3.41	0.00	3.36	0.05	0.36	0.056
1192	CLIC1	GO_GLUTATHIONE_METABOLIC_PROCESS	9.36	0.03	9.28	0.03	0.11	0.079
1193	CLIC2	GO_GLUTATHIONE_METABOLIC_PROCESS	2.85	0.14	2.94	0.09	0.54	-0.091
9022	CLIC3	GO_GLUTATHIONE_METABOLIC_PROCESS	5.78	0.06	5.65	0.29	0.63	0.134
25932	CLIC4	GO_GLUTATHIONE_METABOLIC_PROCESS	9.80	0.07	9.70	0.21	0.60	0.104
53405	CLIC5	GO_GLUTATHIONE_METABOLIC_PROCESS	2.63	0.05	2.59	0.08	0.67	0.035
54102	CLIC6	GO_GLUTATHIONE_METABOLIC_PROCESS	2.77	0.08	2.93	0.04	0.16	-0.164
55748	CNDP2	GO_GLUTATHIONE_METABOLIC_PROCESS	8.68	0.07	9.09	0.02	0.06	-0.409
1373	CPS1	GO_GLUTAMINE_FAMILY_AMINO_ACID_BIOSYNTHETIC_PROCESS, GO_GLUTAMINE_FAMILY_AMINO_ACID_METABOLIC_PROCESS, GO_GLUTAMINE_METABOLIC_PROCESS	7.33	0.05	6.99	0.14	0.15	0.340
1497	CTNS	GO_GLUTATHIONE_METABOLIC_PROCESS	6.22	0.02	5.93	0.12	0.18	0.291
1503	CTPS1	GO_GLUTAMINE_FAMILY_AMINO_ACID_METABOLIC_PROCESS, GO_GLUTAMINE_METABOLIC_PROCESS	6.42	0.12	6.43	0.14	0.94	-0.011
56474	CTPS2	GO_GLUTAMINE_FAMILY_AMINO_ACID_METABOLIC_PROCESS, GO_GLUTAMINE_METABOLIC_PROCESS	6.48	0.07	6.65	0.06	0.12	-0.177
1610	DAO	GO_GLUTAMINE_FAMILY_AMINO_ACID_CATABOLIC_PROCESS, GO_GLUTAMINE_FAMILY_AMINO_ACID_METABOLIC_PROCESS	4.41	0.01	4.42	0.25	0.98	-0.004
23576	DDAH1	GLUTAMINE_FAMILY_AMINO_ACID_METABOLIC_PROCESS, GO_GLUTAMINE_FAMILY_AMINO_ACID_CATABOLIC_PROCESS, GO_GLUTAMINE_FAMILY_AMINO_ACID_METABOLIC_PROCESS	6.22	0.10	6.76	0.16	0.08	-0.543
23564	DDAH2	GLUTAMINE_FAMILY_AMINO_ACID_METABOLIC_PROCESS, GO_GLUTAMINE_FAMILY_AMINO_ACID_CATABOLIC_PROCESS, GO_GLUTAMINE_FAMILY_AMINO_ACID_METABOLIC_PROCESS	5.98	0.00	6.04	0.20	0.77	-0.054
1800	DPEP1	GO_GLUTATHIONE_METABOLIC_PROCESS	5.89	0.00	5.73	0.31	0.60	0.160
9521	EEF1E1	GO_GLUTATHIONE_METABOLIC_PROCESS	5.89	0.06	5.78	0.16	0.50	0.111
1937	EEF1G	GO_GLUTATHIONE_METABOLIC_PROCESS	11.03	0.05	11.17	0.01	0.14	-0.134

23474	ETHE1	GO_GLUTATHIONE_METABOLIC_PROCESS	6.87	0.03	6.82	0.05	0.40	0.048
2184	FAH	GO_GLUTAMINE_FAMILY_AMINO_ACID_CATABOLIC_PROCESS, GO_GLUTAMINE_FAMILY_AMINO_ACID_METABOLIC_PROCESS	7.28	0.12	6.98	0.07	0.12	0.300
2356	FPGS	GLUTAMINE_FAMILY_AMINO_ACID_METABOLIC_PROCESS, GO_GLUTAMATE_METABOLIC_PROCESS, GO_GLUTAMINE_FAMILY_AMINO_ACID_CATABOLIC_PROCESS, GO_GLUTAMINE_FAMILY_AMINO_ACID_METABOLIC_PROCESS	7.04	0.08	6.87	0.15	0.32	0.173
10841	FTCD	GO_GLUTAMATE_METABOLIC_PROCESS, GO_GLUTAMINE_FAMILY_AMINO_ACID_METABOLIC_PROCESS	6.18	0.10	6.07	0.18	0.58	0.103
2539	G6PD	GO_GLUTATHIONE_METABOLIC_PROCESS	9.20	0.18	9.27	0.06	0.67	-0.075
2571	GAD1	GLUTAMINE_FAMILY_AMINO_ACID_METABOLIC_PROCESS, GO_GLUTAMATE_METABOLIC_PROCESS, GO_GLUTAMINE_FAMILY_AMINO_ACID_CATABOLIC_PROCESS, GO_GLUTAMINE_FAMILY_AMINO_ACID_METABOLIC_PROCESS	5.20	0.04	5.20	0.11	0.98	-0.002
2572	GAD2	GLUTAMINE_FAMILY_AMINO_ACID_METABOLIC_PROCESS, GO_GLUTAMATE_METABOLIC_PROCESS, GO_GLUTAMINE_FAMILY_AMINO_ACID_CATABOLIC_PROCESS, GO_GLUTAMINE_FAMILY_AMINO_ACID_METABOLIC_PROCESS	3.20	0.01	3.13	0.10	0.51	0.067
2729	GCLC	GLUTAMINE_FAMILY_AMINO_ACID_METABOLIC_PROCESS, GO_GLUTAMATE_METABOLIC_PROCESS, GO_GLUTAMINE_FAMILY_AMINO_ACID_METABOLIC_PROCESS, GO_GLUTATHIONE_METABOLIC_PROCESS	5.11	0.02	5.27	0.14	0.36	-0.160
2730	GCLM	GLUTAMINE_FAMILY_AMINO_ACID_METABOLIC_PROCESS, GO_GLUTAMATE_METABOLIC_PROCESS, GO_GLUTAMINE_FAMILY_AMINO_ACID_METABOLIC_PROCESS, GO_GLUTATHIONE_METABOLIC_PROCESS	7.29	0.03	7.31	0.13	0.86	-0.022
54332	GDAP1	GO_GLUTATHIONE_METABOLIC_PROCESS	4.46	0.01	4.38	0.32	0.79	0.079
78997	GDAP1L1	GO_GLUTATHIONE_METABOLIC_PROCESS	5.82	0.09	5.73	0.18	0.61	0.092
2673	GFPT1	GO_GLUTAMINE_FAMILY_AMINO_ACID_METABOLIC_PROCESS, GO_GLUTAMINE_METABOLIC_PROCESS	9.09	0.00	9.76	0.18	0.12	-0.667
9945	GFPT2	GO_GLUTAMINE_FAMILY_AMINO_ACID_METABOLIC_PROCESS, GO_GLUTAMINE_METABOLIC_PROCESS	5.99	0.20	5.85	0.08	0.49	0.138
79017	GGCT	GO_GLUTATHIONE_METABOLIC_PROCESS	6.49	0.13	6.42	0.28	0.80	0.068
8836	GGH	GO_GLUTAMINE_FAMILY_AMINO_ACID_METABOLIC_PROCESS, GO_GLUTAMINE_METABOLIC_PROCESS	7.00	0.05	6.99	0.11	0.91	0.012
2678	GGT1	GO_GLUTAMATE_METABOLIC_PROCESS, GO_GLUTAMINE_FAMILY_AMINO_ACID_METABOLIC_PROCESS, GO_GLUTATHIONE_METABOLIC_PROCESS	8.40	0.05	8.52	0.10	0.31	-0.117
728441	GGT2	GO_GLUTATHIONE_METABOLIC_PROCESS	8.40	0.05	8.52	0.10	0.31	-0.117
2679	GGT3P	GO_GLUTATHIONE_METABOLIC_PROCESS	7.89	0.04	8.01	0.02	0.09	-0.114
2687	GGT5	GO_GLUTATHIONE_METABOLIC_PROCESS	6.29	0.01	6.33	0.13	0.68	-0.048

124975	GGT6	GO_GLUTATHIONE_METABOLIC_PROCESS	5.64	0.07	5.63	0.12	0.93	0.010
2686	GGT7	GO_GLUTATHIONE_METABOLIC_PROCESS	6.26	0.12	6.39	0.15	0.44	-0.130
2681	GGTA1P	GO_GLUTATHIONE_METABOLIC_PROCESS	3.86	0.00	3.85	0.04	0.85	0.007
92086	GGTLC1	GO_GLUTATHIONE_METABOLIC_PROCESS	8.40	0.05	8.52	0.10	0.31	-0.117
91227	GGTLC2	GO_GLUTATHIONE_METABOLIC_PROCESS	8.40	0.05	8.52	0.10	0.31	-0.117
2739	GLO1	GO_GLUTATHIONE_METABOLIC_PROCESS	7.96	0.14	8.30	0.12	0.12	-0.341
51022	GLRX2	GO_GLUTATHIONE_METABOLIC_PROCESS	6.04	0.06	6.02	0.02	0.76	0.017
2744	GLS	GO_GLUTAMATE_METABOLIC_PROCESS, GO_GLUTAMINE_FAMILY_AMINO_ACID_BIOSYNTHETIC_PROCESS, GO_GLUTAMINE_FAMILY_AMINO_ACID_CATABOLIC_PROCESS, GO_GLUTAMINE_FAMILY_AMINO_ACID_METABOLIC_PROCESS, GO_GLUTAMINE_METABOLIC_PROCESS	7.37	0.07	7.75	0.02	0.07	-0.380
27165	GLS2	GO_GLUTAMATE_METABOLIC_PROCESS, GO_GLUTAMINE_FAMILY_AMINO_ACID_BIOSYNTHETIC_PROCESS, GO_GLUTAMINE_FAMILY_AMINO_ACID_CATABOLIC_PROCESS, GO_GLUTAMINE_FAMILY_AMINO_ACID_METABOLIC_PROCESS, GO_GLUTAMINE_METABOLIC_PROCESS	4.43	0.11	4.48	0.13	0.73	-0.049
2746	GLUD1	GLUTAMINE_FAMILY_AMINO_ACID_METABOLIC_PROCESS, GO_GLUTAMATE_METABOLIC_PROCESS, GO_GLUTAMINE_FAMILY_AMINO_ACID_BIOSYNTHETIC_PROCESS, GO_GLUTAMINE_FAMILY_AMINO_ACID_CATABOLIC_PROCESS, GO_GLUTAMINE_FAMILY_AMINO_ACID_METABOLIC_PROCESS, GO_GLUTAMINE_METABOLIC_PROCESS	8.94	0.05	8.86	0.04	0.23	0.085
2747	GLUD2	GLUTAMINE_FAMILY_AMINO_ACID_METABOLIC_PROCESS, GO_GLUTAMATE_METABOLIC_PROCESS, GO_GLUTAMINE_FAMILY_AMINO_ACID_BIOSYNTHETIC_PROCESS, GO_GLUTAMINE_FAMILY_AMINO_ACID_CATABOLIC_PROCESS, GO_GLUTAMINE_FAMILY_AMINO_ACID_METABOLIC_PROCESS	4.70	0.04	4.60	0.23	0.64	0.102
2752	GLUL	GO_GLUTAMATE_METABOLIC_PROCESS, GO_GLUTAMINE_FAMILY_AMINO_ACID_BIOSYNTHETIC_PROCESS, GO_GLUTAMINE_FAMILY_AMINO_ACID_CATABOLIC_PROCESS, GO_GLUTAMINE_FAMILY_AMINO_ACID_METABOLIC_PROCESS, GO_GLUTAMINE_METABOLIC_PROCESS	5.14	0.07	4.85	0.11	0.11	0.289
8833	GMPS	GO_GLUTAMINE_FAMILY_AMINO_ACID_METABOLIC_PROCESS, GO_GLUTAMINE_METABOLIC_PROCESS	8.54	0.10	8.39	0.19	0.45	0.152
2805	GOT1	GO_GLUTAMATE_METABOLIC_PROCESS, GO_GLUTAMINE_FAMILY_AMINO_ACID_CATABOLIC_PROCESS, GO_GLUTAMINE_FAMILY_AMINO_ACID_METABOLIC_PROCESS	8.41	0.01	8.40	0.05	0.78	0.012
2806	GOT2	GO_GLUTAMATE_METABOLIC_PROCESS, GO_GLUTAMINE_FAMILY_AMINO_ACID_CATABOLIC_PROCESS,	8.20	0.08	8.41	0.10	0.15	-0.207

		GO_GLUTAMINE_FAMILY_AMINO_ACID_METABOLIC_PROCESS						
2876	GPX1	GO_GLUTATHIONE_METABOLIC_PROCESS	7.58	0.01	7.78	0.04	0.09	-0.206
2936	GSR	GO_GLUTATHIONE_METABOLIC_PROCESS	8.04	0.03	7.97	0.03	0.18	0.068
2937	GSS	GO_GLUTATHIONE_METABOLIC_PROCESS	7.43	0.02	7.75	0.01	0.02	-0.321
2938	GSTA1	GO_GLUTATHIONE_METABOLIC_PROCESS	2.95	0.03	2.94	0.04	0.88	0.006
2939	GSTA2	GO_GLUTATHIONE_METABOLIC_PROCESS	3.04	0.09	2.85	0.05	0.14	0.193
2940	GSTA3	GO_GLUTATHIONE_METABOLIC_PROCESS	3.11	0.16	2.86	0.25	0.37	0.250
2941	GSTA4	GO_GLUTATHIONE_METABOLIC_PROCESS	5.19	0.01	5.24	0.04	0.30	-0.051
221357	GSTA5	GO_GLUTATHIONE_METABOLIC_PROCESS	3.38	0.02	3.33	0.04	0.33	0.048
373156	GSTK1	GO_GLUTATHIONE_METABOLIC_PROCESS	7.15	0.03	7.17	0.04	0.64	-0.021
2944	GSTM1	GO_GLUTATHIONE_METABOLIC_PROCESS	5.23	0.13	5.56	0.05	0.14	-0.327
2946	GSTM2	GO_GLUTATHIONE_METABOLIC_PROCESS	4.82	0.04	5.16	0.05	0.02	-0.340
2947	GSTM3	GO_GLUTATHIONE_METABOLIC_PROCESS	5.83	0.02	6.13	0.18	0.24	-0.303
2948	GSTM4	GO_GLUTATHIONE_METABOLIC_PROCESS	7.17	0.11	7.73	0.11	0.04	-0.560
2949	GSTM5	GO_GLUTATHIONE_METABOLIC_PROCESS	4.72	0.04	4.93	0.09	0.14	-0.210
9446	GSTO1	GO_GLUTATHIONE_METABOLIC_PROCESS	6.10	0.03	6.08	0.00	0.54	0.020
119391	GSTO2	GO_GLUTATHIONE_METABOLIC_PROCESS	3.88	0.08	4.03	0.13	0.33	-0.150
2950	GSTP1	GO_GLUTATHIONE_METABOLIC_PROCESS	8.45	0.07	8.73	0.02	0.11	-0.284
2952	GSTT1	GO_GLUTATHIONE_METABOLIC_PROCESS	8.23	0.26	8.36	0.04	0.62	-0.127
2953	GSTT2	GO_GLUTATHIONE_METABOLIC_PROCESS	5.56	0.03	5.65	0.11	0.45	-0.090
653689	GSTT2B	GO_GLUTATHIONE_METABOLIC_PROCESS	5.56	0.03	5.65	0.11	0.45	-0.090
2954	GSTZ1	GO_GLUTATHIONE_METABOLIC_PROCESS	5.90	0.01	6.00	0.11	0.44	-0.096
3029	HAGH	GO_GLUTATHIONE_METABOLIC_PROCESS	7.29	0.06	7.42	0.08	0.22	-0.129
3034	HAL	GO_GLUTAMATE_METABOLIC_PROCESS, GO_GLUTAMINE_FAMILY_AMINO_ACID_METABOLIC_PROCESS	4.07	0.17	3.97	0.15	0.63	0.091
3417	IDH1	GO_GLUTATHIONE_METABOLIC_PROCESS	7.42	0.03	7.45	0.12	0.83	-0.024
51557	LGSN	GO_GLUTAMINE_FAMILY_AMINO_ACID_BIOSYNTHETIC_PROCESS, GO_GLUTAMINE_FAMILY_AMINO_ACID_METABOLIC_PROCESS, GO_GLUTAMINE_METABOLIC_PROCESS	2.18	0.01	2.17	0.12	0.91	0.011
4204	MECP2	GO_GLUTAMINE_FAMILY_AMINO_ACID_METABOLIC_PROCESS, GO_GLUTAMINE_METABOLIC_PROCESS	6.15	0.02	6.25	0.06	0.28	-0.091
4257	MGST1	GO_GLUTATHIONE_METABOLIC_PROCESS	7.45	0.00	7.28	0.14	0.32	0.174
4258	MGST2	GO_GLUTATHIONE_METABOLIC_PROCESS	5.37	0.08	5.35	0.05	0.75	0.024
10588	MTHFS	GO_GLUTAMATE_METABOLIC_PROCESS, GO_GLUTAMINE_FAMILY_AMINO_ACID_METABOLIC_PROCESS	5.37	0.16	5.69	0.07	0.18	-0.321
162417	NAGS	GO_GLUTAMATE_METABOLIC_PROCESS, GO_GLUTAMINE_FAMILY_AMINO_ACID_BIOSYNTHETIC_PROCESS, GO_GLUTAMINE_FAMILY_AMINO_ACID_METABOLIC_PROCESS	6.54	0.08	6.51	0.15	0.81	0.033
9027	NAT8	GO_GLUTATHIONE_METABOLIC_PROCESS	4.61	0.02	4.59	0.18	0.86	0.028
56954	NIT2	GO_GLUTAMINE_FAMILY_AMINO_ACID_METABOLIC_PROCESS, GO_GLUTAMINE_METABOLIC_PROCESS	6.94	0.00	7.07	0.02	0.09	-0.123
4842	NOS1	GO_GLUTAMINE_FAMILY_AMINO_ACID_CATABOLIC_PROCESS, GO_GLUTAMINE_FAMILY_AMINO_ACID_METABOLIC_PROCESS	3.96	0.20	3.93	0.24	0.91	0.030

4843	NOS2	GO_GLUTAMINE_FAMILY_AMINO_ACID_CATABOLIC_PROCESS, GO_GLUTAMINE_FAMILY_AMINO_ACID_METABOLIC_PROCESS	4.97	0.01	4.84	0.15	0.45	0.124
4846	NOS3	GO_GLUTAMINE_FAMILY_AMINO_ACID_CATABOLIC_PROCESS, GO_GLUTAMINE_FAMILY_AMINO_ACID_METABOLIC_PROCESS	5.90	0.07	5.81	0.19	0.62	0.091
122945	NOXRED1	GO_GLUTAMINE_FAMILY_AMINO_ACID_BIOSYNTHETIC_PROCESS, GO_GLUTAMINE_FAMILY_AMINO_ACID_METABOLIC_PROCESS	3.67	0.07	3.74	0.22	0.73	-0.071
4942	OAT	GO_GLUTAMATE_METABOLIC_PROCESS, GO_GLUTAMINE_FAMILY_AMINO_ACID_BIOSYNTHETIC_PROCESS, GO_GLUTAMINE_FAMILY_AMINO_ACID_CATABOLIC_PROCESS, GO_GLUTAMINE_FAMILY_AMINO_ACID_METABOLIC_PROCESS	6.70	0.06	6.69	0.18	0.95	0.010
26873	OPLAH	GO_GLUTATHIONE_METABOLIC_PROCESS	6.40	0.02	6.30	0.21	0.64	0.094
5009	OTC	GO_GLUTAMINE_FAMILY_AMINO_ACID_BIOSYNTHETIC_PROCESS, GO_GLUTAMINE_FAMILY_AMINO_ACID_METABOLIC_PROCESS	2.66	0.00	2.53	0.18	0.50	0.124
5198	PFAS	GO_GLUTAMINE_FAMILY_AMINO_ACID_METABOLIC_PROCESS, GO_GLUTAMINE_METABOLIC_PROCESS	6.83	0.03	6.77	0.03	0.20	0.058
26227	PHGDH	GO_GLUTAMINE_FAMILY_AMINO_ACID_METABOLIC_PROCESS, GO_GLUTAMINE_METABOLIC_PROCESS	4.67	0.00	4.66	0.08	0.86	0.013
5471	PPAT	GO_GLUTAMINE_FAMILY_AMINO_ACID_CATABOLIC_PROCESS, GO_GLUTAMINE_FAMILY_AMINO_ACID_METABOLIC_PROCESS, GO_GLUTAMINE_METABOLIC_PROCESS	6.83	0.05	6.99	0.24	0.53	-0.156
5625	PRODH	GO_GLUTAMATE_METABOLIC_PROCESS, GO_GLUTAMINE_FAMILY_AMINO_ACID_CATABOLIC_PROCESS, GO_GLUTAMINE_FAMILY_AMINO_ACID_METABOLIC_PROCESS	6.50	0.05	6.29	0.16	0.29	0.211
58510	PRODH2	GO_GLUTAMATE_METABOLIC_PROCESS, GO_GLUTAMINE_FAMILY_AMINO_ACID_CATABOLIC_PROCESS, GO_GLUTAMINE_FAMILY_AMINO_ACID_METABOLIC_PROCESS	5.61	0.01	5.61	0.17	0.99	0.002
5831	PYCR1	GLUTAMINE_FAMILY_AMINO_ACID_METABOLIC_PROCESS, GO_GLUTAMINE_FAMILY_AMINO_ACID_BIOSYNTHETIC_PROCESS, GO_GLUTAMINE_FAMILY_AMINO_ACID_METABOLIC_PROCESS	8.49	0.06	8.68	0.07	0.09	-0.190
29920	PYCR2	GO_GLUTAMINE_FAMILY_AMINO_ACID_BIOSYNTHETIC_PROCESS, GO_GLUTAMINE_FAMILY_AMINO_ACID_METABOLIC_PROCESS	6.87	0.12	6.92	0.08	0.63	-0.057
65263	PYCR3	GO_GLUTAMINE_FAMILY_AMINO_ACID_BIOSYNTHETIC_PROCESS, GO_GLUTAMINE_FAMILY_AMINO_ACID_METABOLIC_PROCESS	6.34	0.03	6.28	0.07	0.39	0.061
23409	SIRT4	GO_GLUTAMINE_FAMILY_AMINO_ACID_METABOLIC_PROCESS, GO_GLUTAMINE_METABOLIC_PROCESS	4.02	0.17	3.97	0.14	0.77	0.053

6507	SLC1A3	GO_GLUTAMATE_METABOLIC_PROCESS, GO_GLUTAMINE_FAMILY_AMINO_ACID_BIOSYNTHETIC_PROCESS, GO_GLUTAMINE_FAMILY_AMINO_ACID_METABOLIC_PROCESS	4.10	0.06	4.26	0.09	0.19	-0.155
6647	SOD1	GO_GLUTATHIONE_METABOLIC_PROCESS	8.90	0.03	9.06	0.01	0.07	-0.159
6898	TAT	GO_GLUTAMATE_METABOLIC_PROCESS, GO_GLUTAMINE_FAMILY_AMINO_ACID_METABOLIC_PROCESS	3.37	0.11	3.25	0.07	0.32	0.125
131669	UROC1	GO_GLUTAMATE_METABOLIC_PROCESS, GO_GLUTAMINE_FAMILY_AMINO_ACID_METABOLIC_PROCESS	5.30	0.05	5.22	0.14	0.58	0.076

Figure S1:

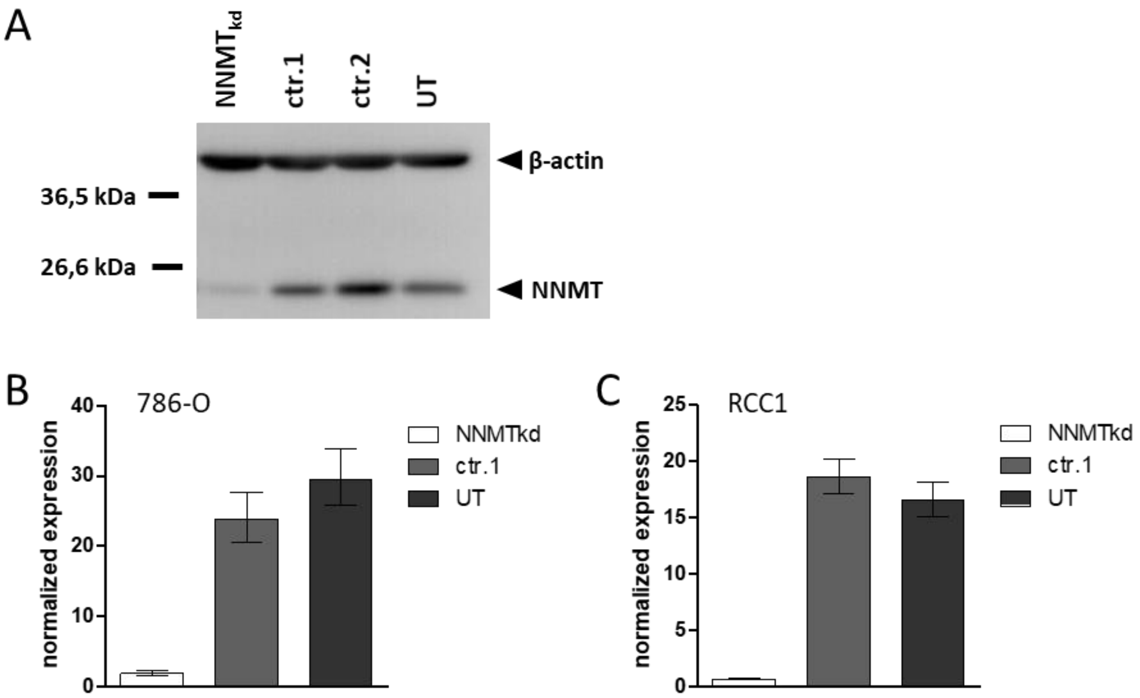

Figure S1: NNMT antibody validation and knockdown efficiency. A, Western blot of 786-O cell lysates (cytosol fraction). 786-O cells were transfected with a siRNA pool targeting NNMT mRNA ($NNMT_{kd}$), with two different non-targeting control siRNA pools (ctr.1 and ctr.2) or left untreated (UT). NNMT and β-actin bands and the respective sizes are indicated in the figure. The proteins were stained subsequently on the same membrane. **B,** NNMT knockdown efficiency in 786-O and **C,** RCC1 cells, assessed by quantitative real-time PCR. NNMT expression levels were normalized to β-actin levels. Shown is one exemplary experiment for each cell line.

Figure S2:

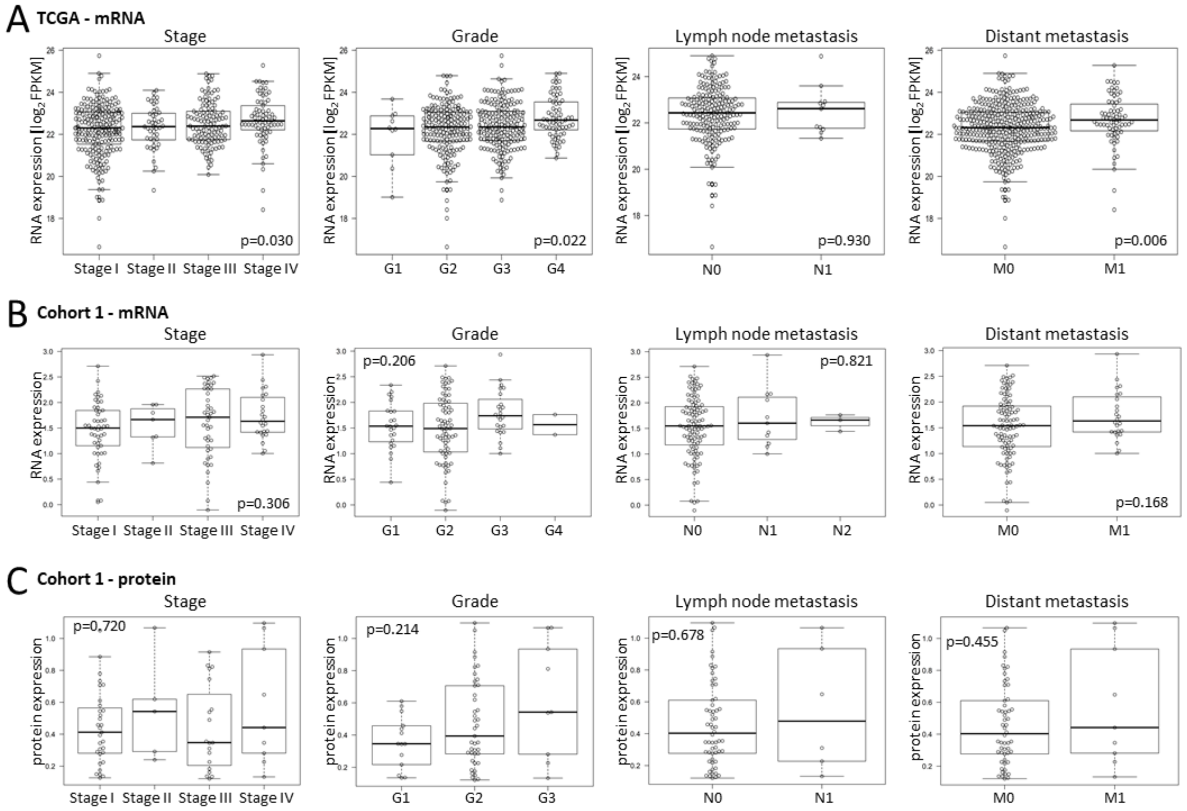

Figure S2: NNMT expression and clinical parameters. A, NNMT mRNA expression and tumor stage, grade, lymph node metastasis, and distant metastasis state at diagnosis in the TCGA KIRC patient cohort. The box plots were generated with the boxplot function in R using default parameters. Kruskal Wallis p-values are given in the plots. **B,** NNMT mRNA and **C,** NNMT protein expression and clinical parameters in patient cohort 1.

Figure S3:

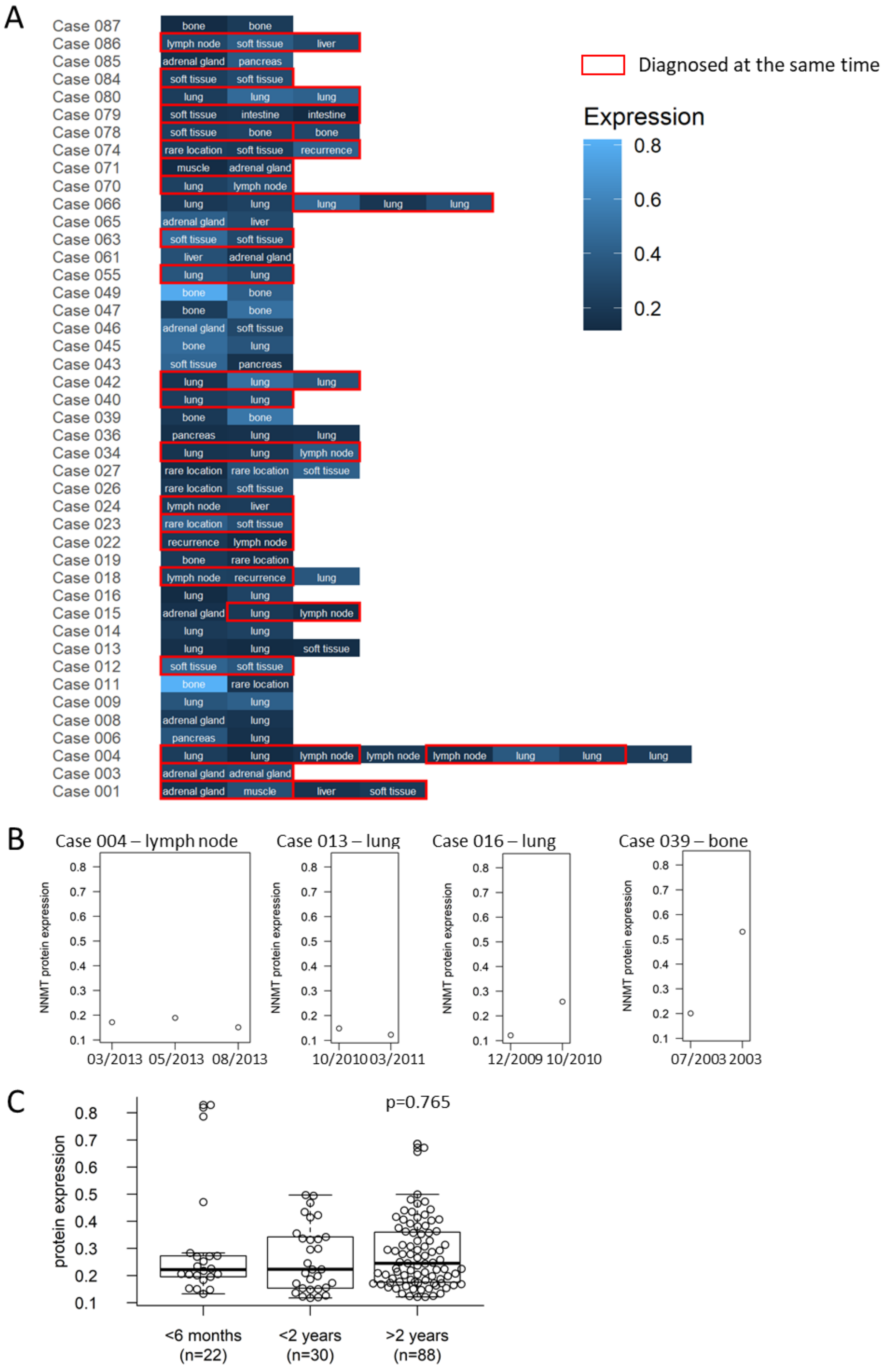

Figure S3: NNMT expression in patients with multiple metastases. A, NNMT expression in multiple metastases from the same patient. **B,** NNMT expression in progressive metastases resected at different time points. **C,** Time-dependent NNMT expression. Metastases were grouped based on their occurrence after initial tumor diagnosis. The difference is not significant (Kruskal-Wallis p-value).

Figure S4:

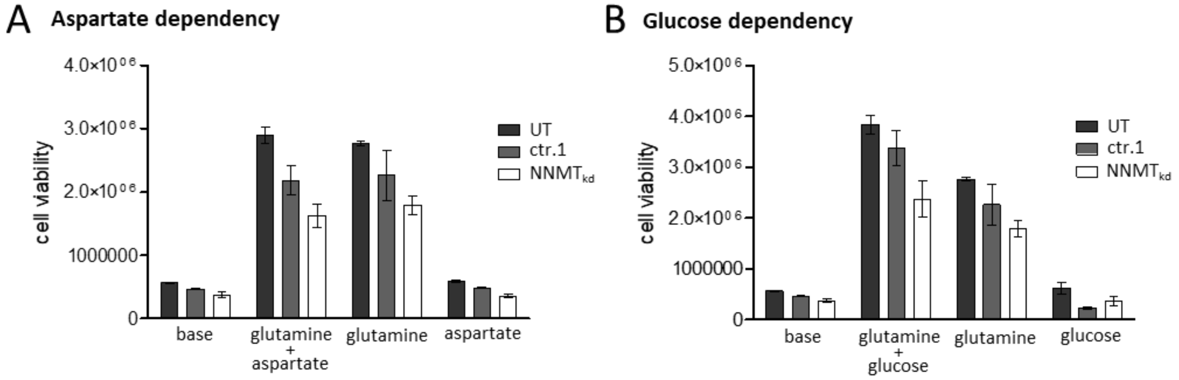

Figure S4: Aspartate and glucose dependency. A, B, Cell viability in $NNMT_{kd}$, non-targeting siRNA control 1 transfected cells (ctr.1) and untreated cells (UT) at different media compositions. Base medium represents RPMI medium without amino acids or glucose (Biomol Cat# R9010-01) supplemented with 10 % FCS. The supplements are indicated at the x-axis. We used 2 mM L-glutamine, 2 g/L glucose and 0.15 mM L-aspartic acid in the experiments, according to the concentrations in standard RPMI medium. The plots show representative data from three independent experiments. The bars represent mean values of 3 replicates +/- SD.

Figure S5:

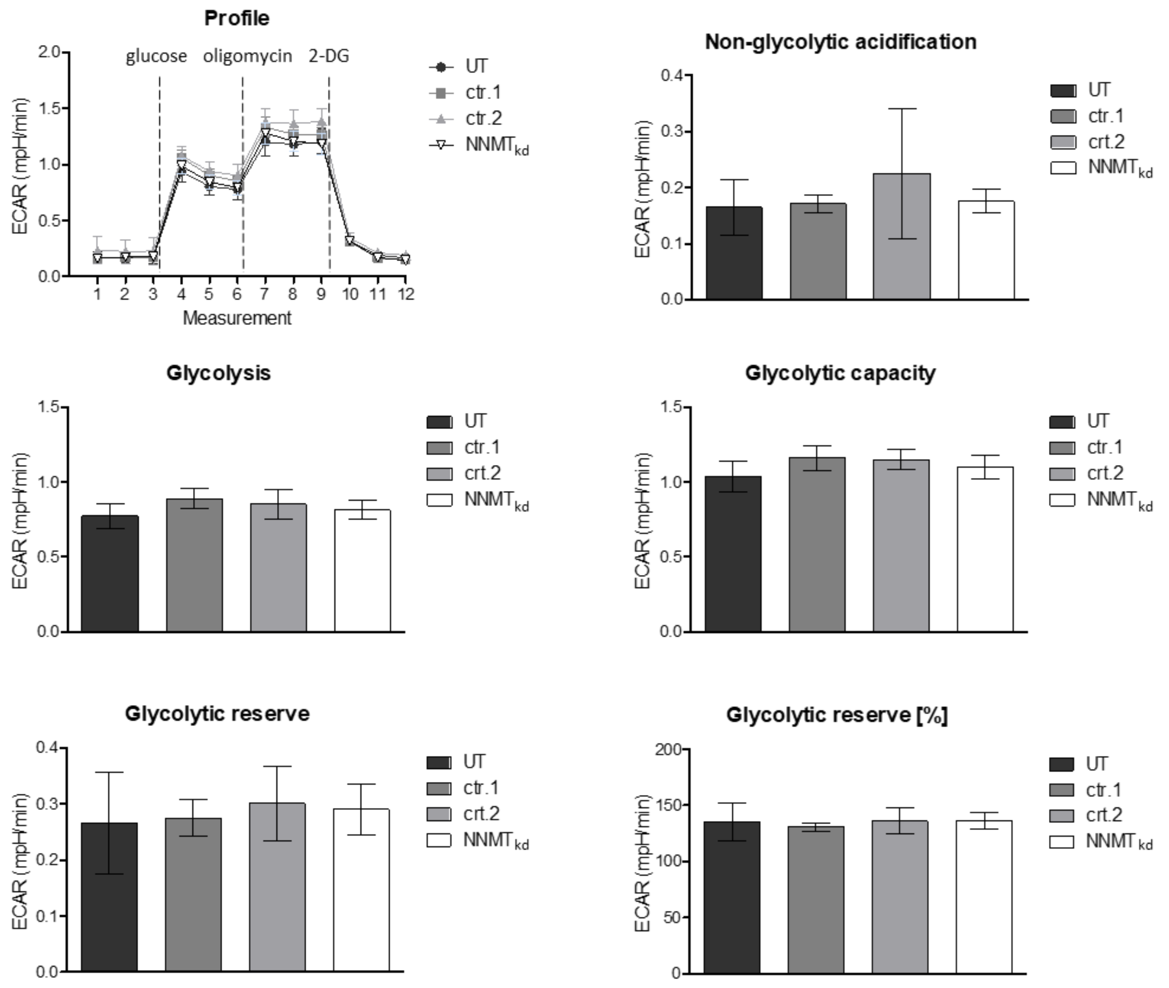

Figure S5: Glycolysis stress test and glycolytic parameters of 786-O cells. Glycolytic activity in 786-O cells measured by extracellular flux analysis with the Glycolysis Stress Test kit (Agilent) in $NNMT_{kd}$ and control cells. Plotted values represent mean values +/- SD of three independent experiments. None of the differences were statistically significant (Student's t test).

Figure S6:

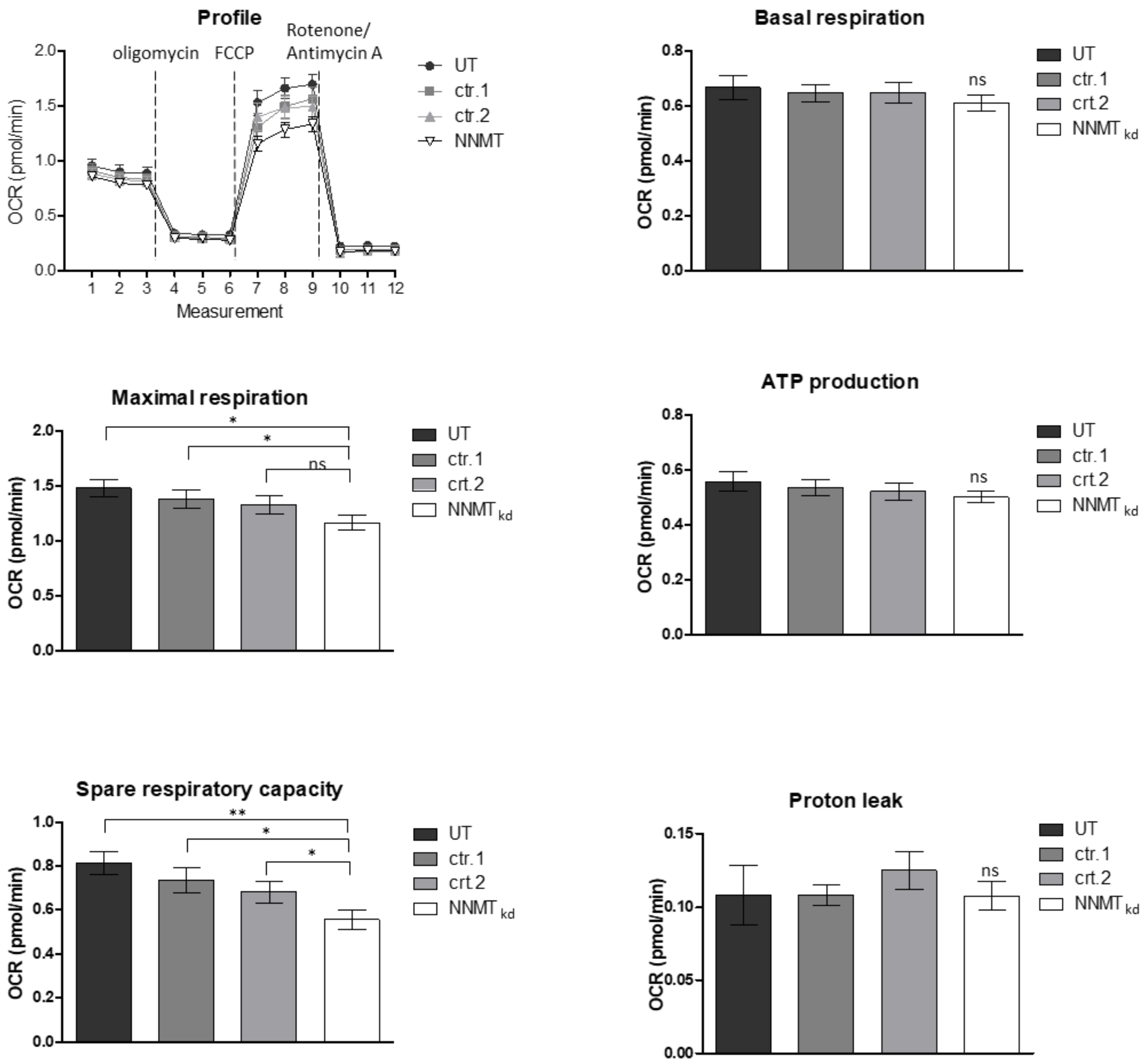

Figure S6: Mitochondrial stress test and mitochondrial parameters in 786-O cells. Mitochondrial activity in 786-O cells was measured by extracellular flux analysis with the Mito Stress Test kit (Agilent) in $NNMT_{kd}$ and control cells. Significant differences are marked by asterisks (Student's t test with Benjamini-Hochberg correction for multiple testing). Significance levels are indicated as follows: ns: not significant; *: $p<0.05$; **: $p<0.01$, ***: $p<0.001$.

Figure S7:

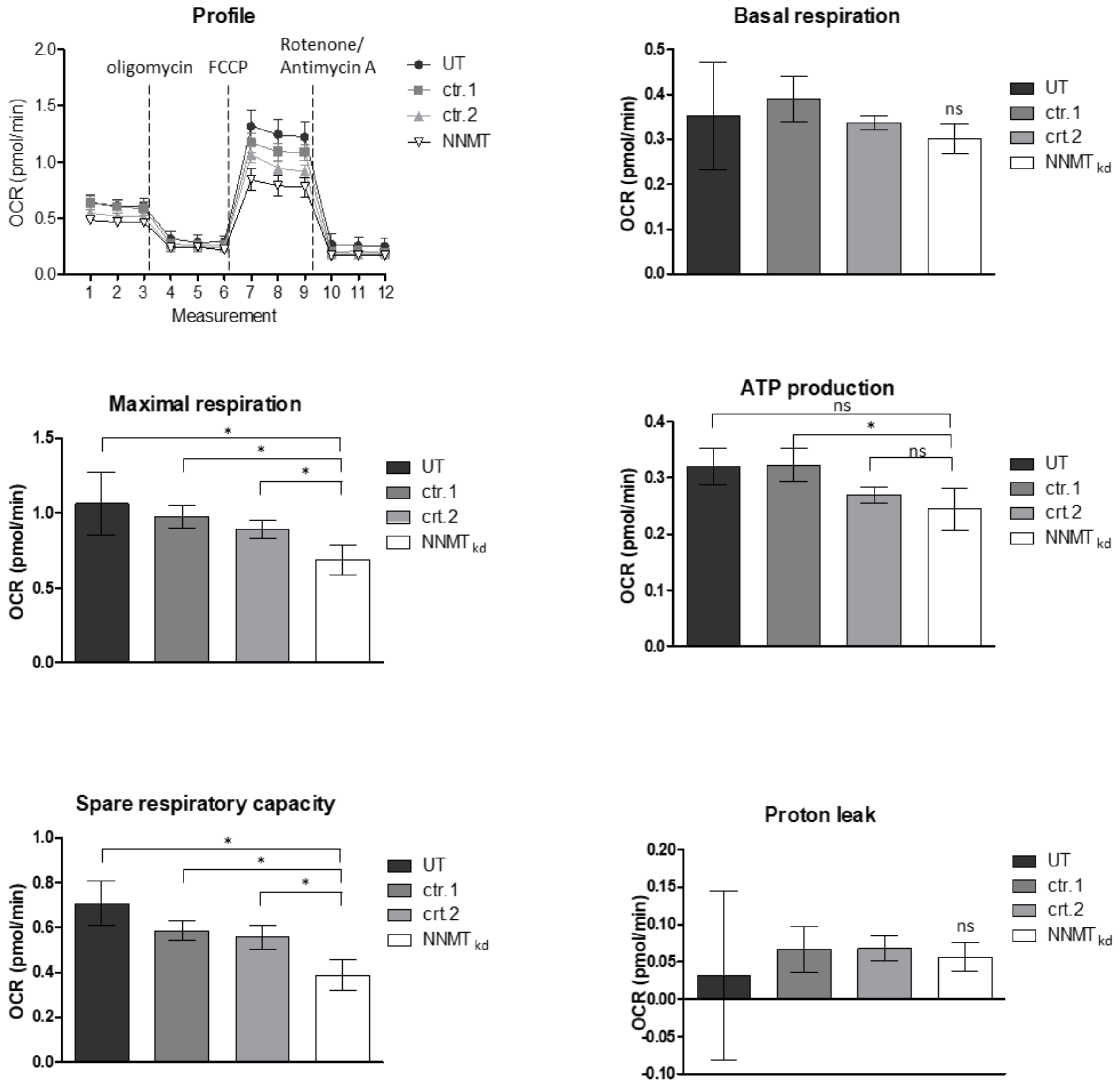

Figure S7: Mitochondrial stress test and mitochondrial parameters in the ccRCC metastasis derived cell culture RCC1. Mitochondrial activity in the primary metastasis-derived culture was measured by extracellular flux analysis with the Mito Stress Test kit (Agilent) in $NNMT_{kd}$ and control cells. Plotted values represent mean values +/- SD of three independent experiments. Significant differences are marked by asterisks (Student's t test with Benjamini-Hochberg correction for multiple testing). Significance levels are indicated as follows: ns: not significant; *: $p<0.05$; **: $p<0.01$, ***: $p<0.001$.

Figure S8:

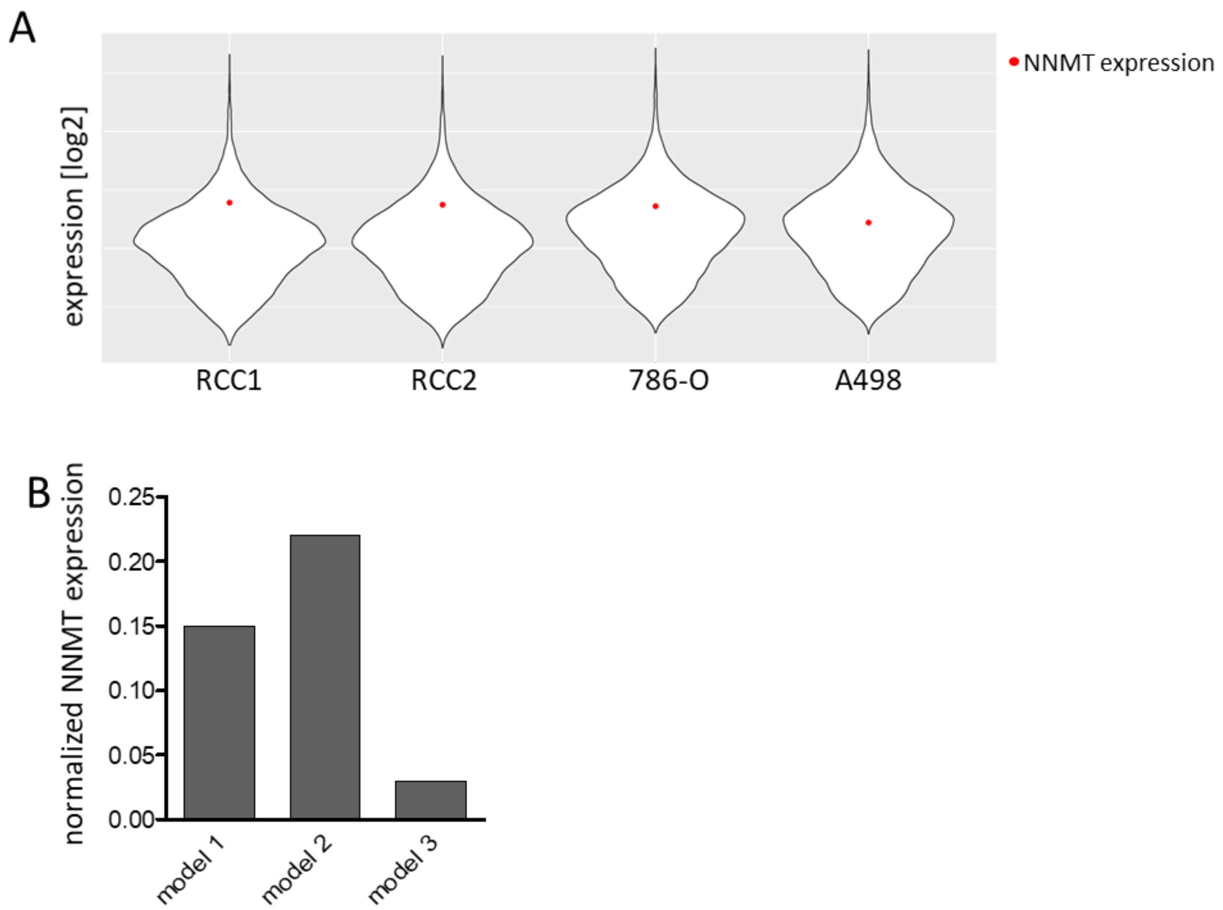

Figure S8: NNMT expression in cell culture models used in the study. A, NNMT mRNA expression in the ccRCC-derived cell lines 786-O and A498, and the primary ccRCC models RCC1 and RCC2. Shown are violin plots of the expression values over all annotated genes covered by the HTA 2.0 Array (Affymetrix). NNMT expression values in the individual cell lines are indicated by the red dots. **B,** NNMT expression in ccRCC air-liquid interface (ALI) models used for the inhibition experiments, measured by quantitative real-time PCR. NNMT expression levels were normalized to β-actin expression.

Figure S9:

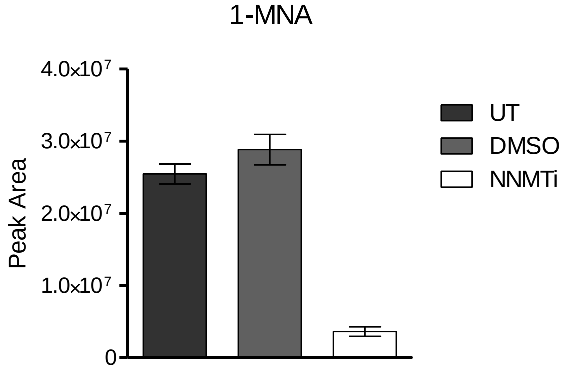

Figure S9: Validation of NNMTi inhibitory activity. 1-methlynicotinamide (1-MNA) levels in untreated 786-O cells (UT), DMSO treated cells, or cells treated with the NNMT inhibitor NNMTi, assessed by LC-QTOF-MS. Values represent means +/- SD of three independent experiments.

Figure S10:

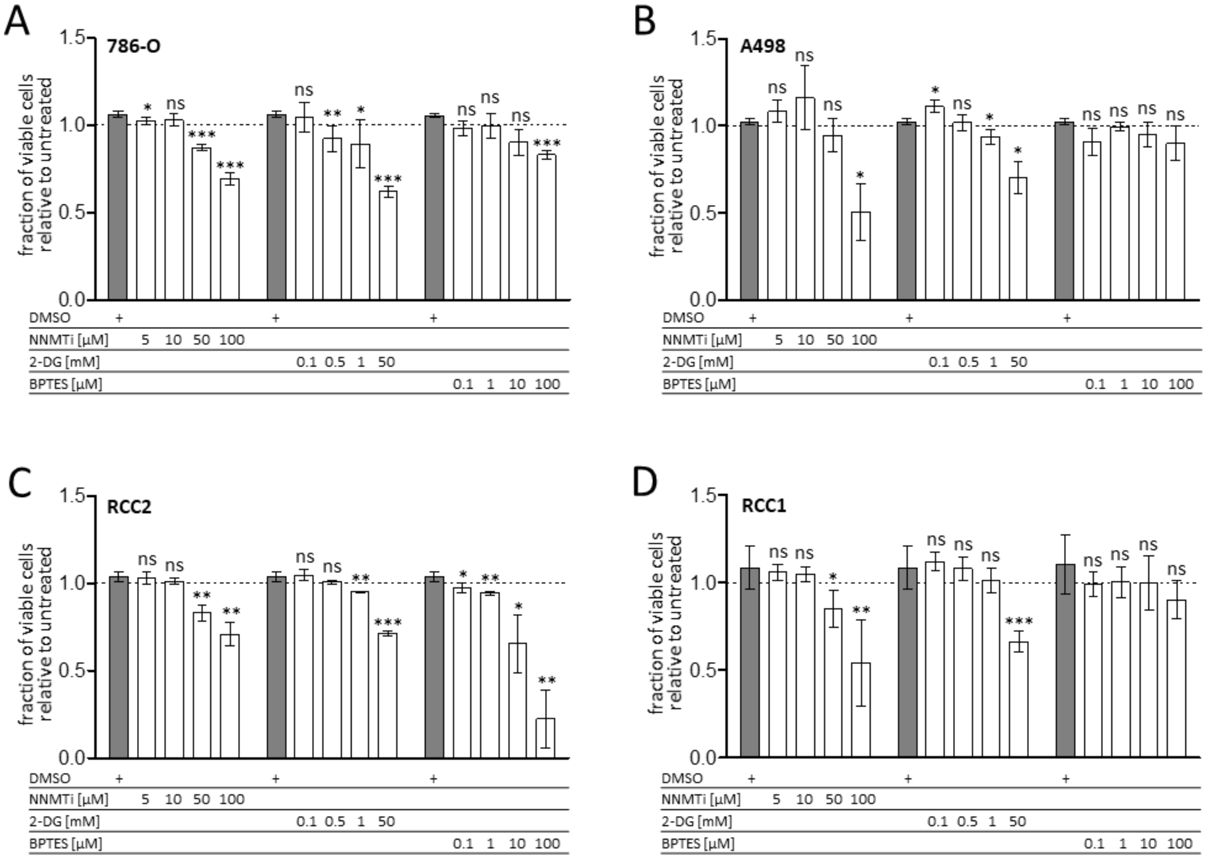

Figure S10: Concentration dependent inhibition of cell viability by NNMTi, 2-DG and BPTES. Shown is the fraction of viable cells relative to untreated control cells for **A,** 786-O, **B,** A498, **C,** RCC2, and **D,** RCC1 cells. Significant differences based on unpaired Student's t test with Benjamini-Hochberg correction for multiple testing are marked by asterisks. Significance levels are indicated as follows: ns: not significant; *: p<0.05; **: p<0.01, ***: p<0.001.

Figure S11:

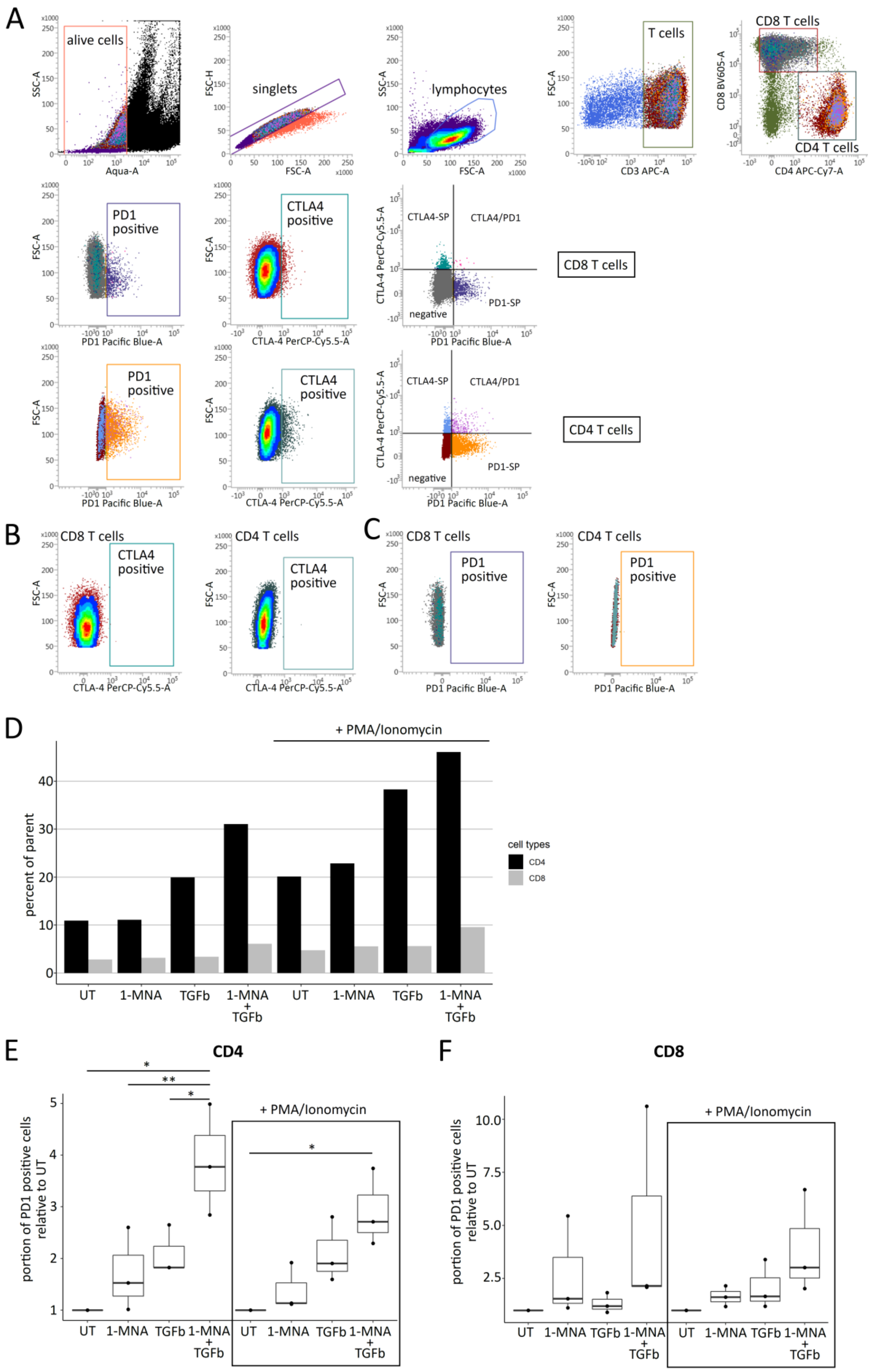
A
alive cells
singlets
lymphocytes
T cells
CD8 T cells
CD4 T cells
Aqua-A
FSC-A
CD3 APC-A
CD4 APC-Cy7-A
CD8 BV605-A
PD1 positive
CTLA4 positive
CTLA4-SP
CTLA4/PD1
PD1-SP
negative
PD1 Pacific Blue-A
CTLA-4 PerCP-Cy5.5-A
B
C
D
+ PMA/Ionomycin
percent of parent
cell types
CD4
CD8
UT
1-MNA
TGFb
1-MNA + TGFb
E
CD4
F
CD8
portion of PD1 positive cells relative to UT

Figure S11: The impact of the metabolite 1-MNA on PD1 expression of activated T cells. Naive T cells were generated from PBMCs of healthy volunteers by stimulation with anti-CD3/anti-CD28 beads in the presence of IL-2 for 48 hours. After bead removal, T cells were treated for 72 hours with 10 mM 1-MNA alone or in combination with 2 ng/mL TGFβ. Half of the cells were stimulated with 20 ng/mL PMA and 1 µg/mL ionomycin for 4 hours prior to analysis. PD1 expression was analyzed by flow cytometry. **A,** Gating strategy for flow cytometry. Dead cells and doublets were excluded before gating on the lymphocyte cell population. An anti-CD3 antibody was used to identify T cells, anti-CD4 and anti-CD8 antibodies were used to select CD4 and CD8 positive T cells, respectively. Anti-CTLA4 and anti-PD1 antibodies were used to identify positive CD4 and CD8 T cells. Fluorochrome minus one (FMO) controls were used to set the gates for **B,** CTLA4 and **C,** PD1 positive cells. **D,** Percent of PD1 single positive (SP) CD4 and CD8 cells shown for one exemplary experiment. **E,** Portion of PD1 positive CD4 T cells. Shown is the data of 3 independent experiments, each normalized to its untreated control sample (UT). Significant differences are indicated in the plot. Significance levels: *: $p<0.05$; **: $p<0.01$. **F,** Portion of PD1 positive CD8 T cells. The differences were not significant.

Figure S12:

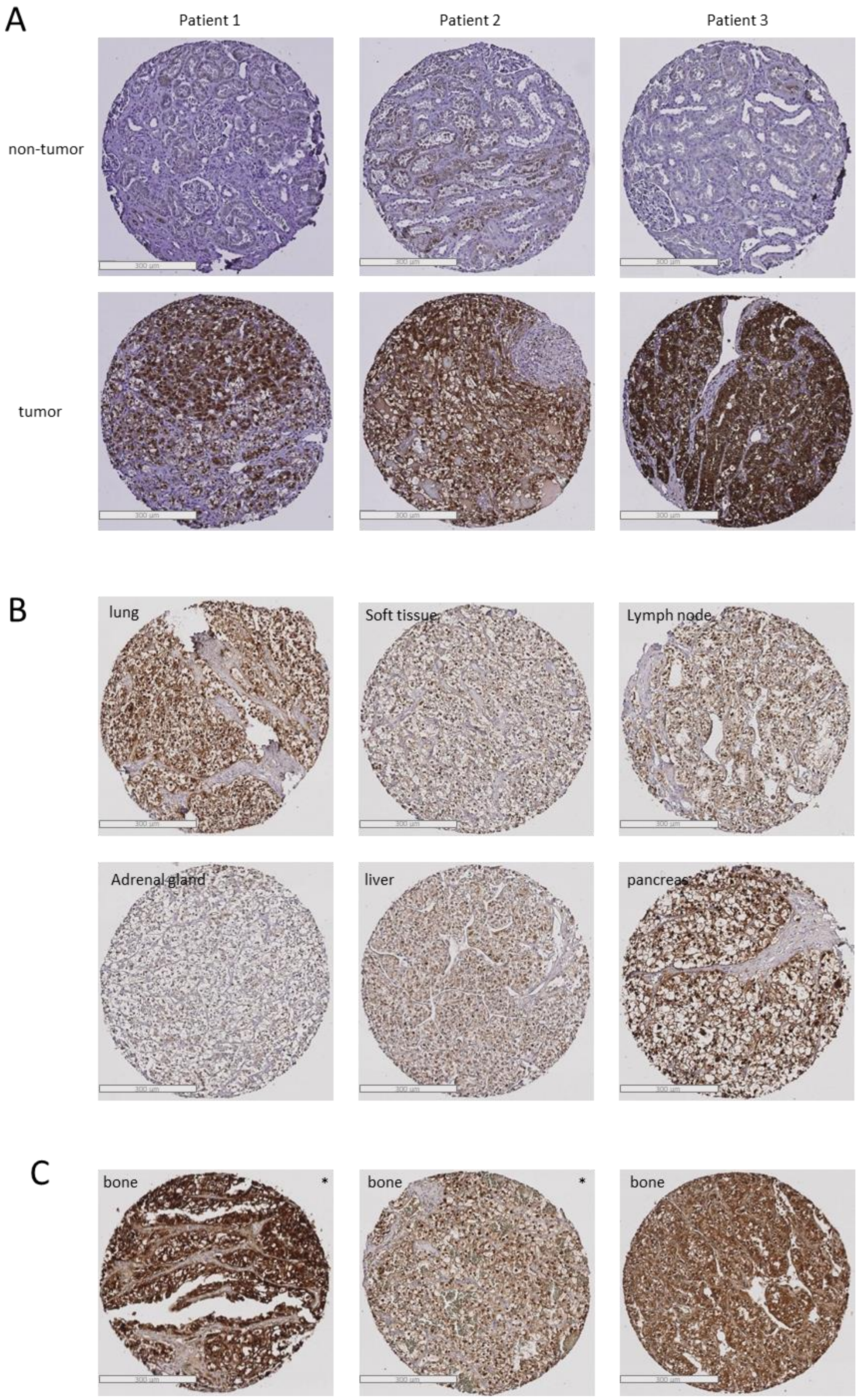

Figure S12: Immunohistochemistry of primary ccRCC tissue, paired non-tumor tissue and ccRCC-derived metastases. A, Immunohistochemical staining of NNMT in paired ccRCC and non-tumor tissues from three patients of cohort 1. **B,** Immunohistochemical staining of NNMT in ccRCC-derived metastases resected from different organs. **C,** Immunohistochemical staining of NNMT in ccRCC-derived bone metastases with different staining intensities.

Figure S13:

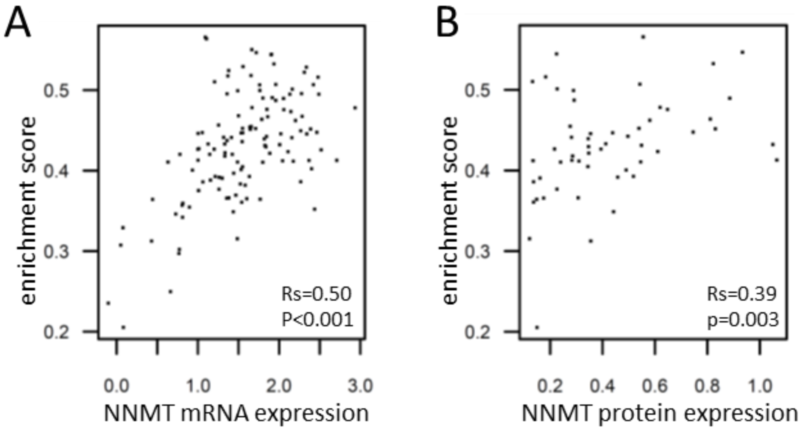

Figure S13: NNMT expression and regulatory T cells in tumor tissue of cohort 1. High NNMT **A,** mRNA and **B,** protein expression is positively correlated with the abundance of regulatory T cells in ccRCC tissue of cohort 1. Regulatory T cells were assessed by gene set enrichment analysis (GSEA) with a signature taken from Chung et al. [4]. The Spearman correlation coefficients (Rs) and corresponding p-values are given in the plots.

References

1. Leuthold P, et al. Comprehensive Metabolomic and Lipidomic Profiling of Human Kidney Tissue: A Platform Comparison. *J Proteome Res.* 2017;16(2):933–944.

2. Metabolite and Chemical Entity Database (METLIN). The Scripps Research Institute, La Jolla, USA. 2017. https://metlin.scripps.edu. Accessed 29 Aug 2017.

3. Molecular Signatures Database. Broad Institute, Cambridge, USA. 2017. http://software.broadinstitute.org/gsea/msigdb. Accessed 6 Oct 2017.

4. Chung W, et al. Single-cell RNA-seq enables comprehensive tumour and immune cell profiling in primary breast cancer. *Nat Commun.* 2017;8:15081.
